# Tuning Charge-Transfer States by Interface Electric Fields

**DOI:** 10.1021/acsami.4c04602

**Published:** 2024-06-06

**Authors:** Anton Kirch, Jakob Wolansky, Shayan Miri Aabi Soflaa, Stephanie Anna Buchholtz, Robert Werberger, Christina Kaiser, Axel Fischer, Karl Leo, Ludvig Edman, Johannes Benduhn, Sebastian Reineke

**Affiliations:** † Dresden Integrated Center for Applied Physics and Photonic Materials (IAPP) and Institute of Applied Physics, Technische Universität Dresden, Nöthnitzer Straße 61, Dresden 01187, Germany; ‡ The Organic Photonics and Electronics Group, Department of Physics, Umeå University, Umeå SE-90187, Sweden

**Keywords:** charge-transfer states, organic p–n junction, exciplex emission, color tuning, interface electric fields

## Abstract

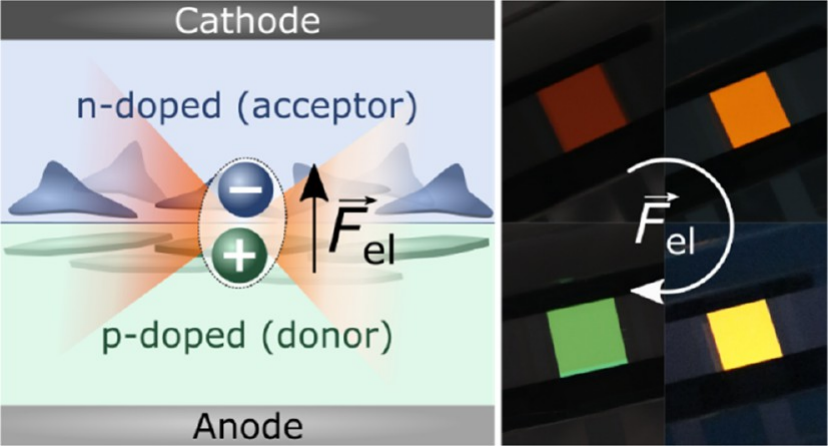

Intermolecular charge-transfer (CT) states are extended excitons with a charge separation on the nanometer scale. Through absorption and emission processes, they couple to the ground state. This property is employed both in light-emitting and light-absorbing devices. Their conception often relies on donor–acceptor (D–A) interfaces, so-called type-II heterojunctions, which usually generate significant electric fields. Several recent studies claim that these fields alter the energetic configuration of the CT states at the interface, an idea holding prospects like multicolor emission from a single emissive interface or shifting the absorption characteristics of a photodetector. Here, we test this hypothesis and contribute to the discussion by presenting a new model system. Through the fabrication of planar organic p-(i-)n junctions, we generate an ensemble of oriented CT states that allows the systematic assessment of electric field impacts. By increasing the thickness of the intrinsic layer at the D–A interface from 0 to 20 nm and by applying external voltages up to 6 V, we realize two different scenarios that controllably tune the intrinsic and extrinsic electric interface fields. By this, we obtain significant shifts of the CT-state peak emission of about 0.5 eV (170 nm from red to green color) from the same D–A material combination. This effect can be explained in a classical electrostatic picture, as the interface electric field alters the potential energy of the electric CT-state dipole. This study illustrates that CT-state energies can be tuned significantly if their electric dipoles are aligned to the interface electric field.

## Introduction

Organic electronic devices mostly rely on the functionalities evoked by thin-film interfaces. If the frontier energy levels of the employed materials are arranged in a donor–acceptor (D–A) configuration (type-II heterojunction), new interface states, so-called intermolecular charge-transfer (CT) states, are formed that couple radiatively to the ground state (GS). These CT excitons are commonly lower in energy than the localized excitons (LE) of the neat bulk materials, which enables the fabrication of organic devices with an optical gap below the neat-material optical gap.^1,2^ For specific D–A combinations, CT states can efficiently generate charge carriers upon illumination or yield significant light emission in electroluminescent (EL) applications. These properties, used in intra- or intermolecular CT-state configurations, are employed in narrowband organic photodetectors (OPDs),^2,3^ light-emitting electrochemical cells (LECs),^4−6^ and organic light-emitting diodes (OLEDs),^7−10^ and govern the open-circuit voltage of organic photovoltaics (OPV).^11−14^ They can also promote triplet harvesting in organic phosphors, used for photoluminescent tags,^15^ oxygen, and wavelength sensors.^16,17^ In the field of light-emitting devices, CT-state radiation is often referred to as exciplex emission.^1^ Regardless of whether the CT-state manifold constitutes the core of the device functionality or may be appointed a culprit for limited performance, its properties are of vital interest to all of the above applications.

**Figure 1:**
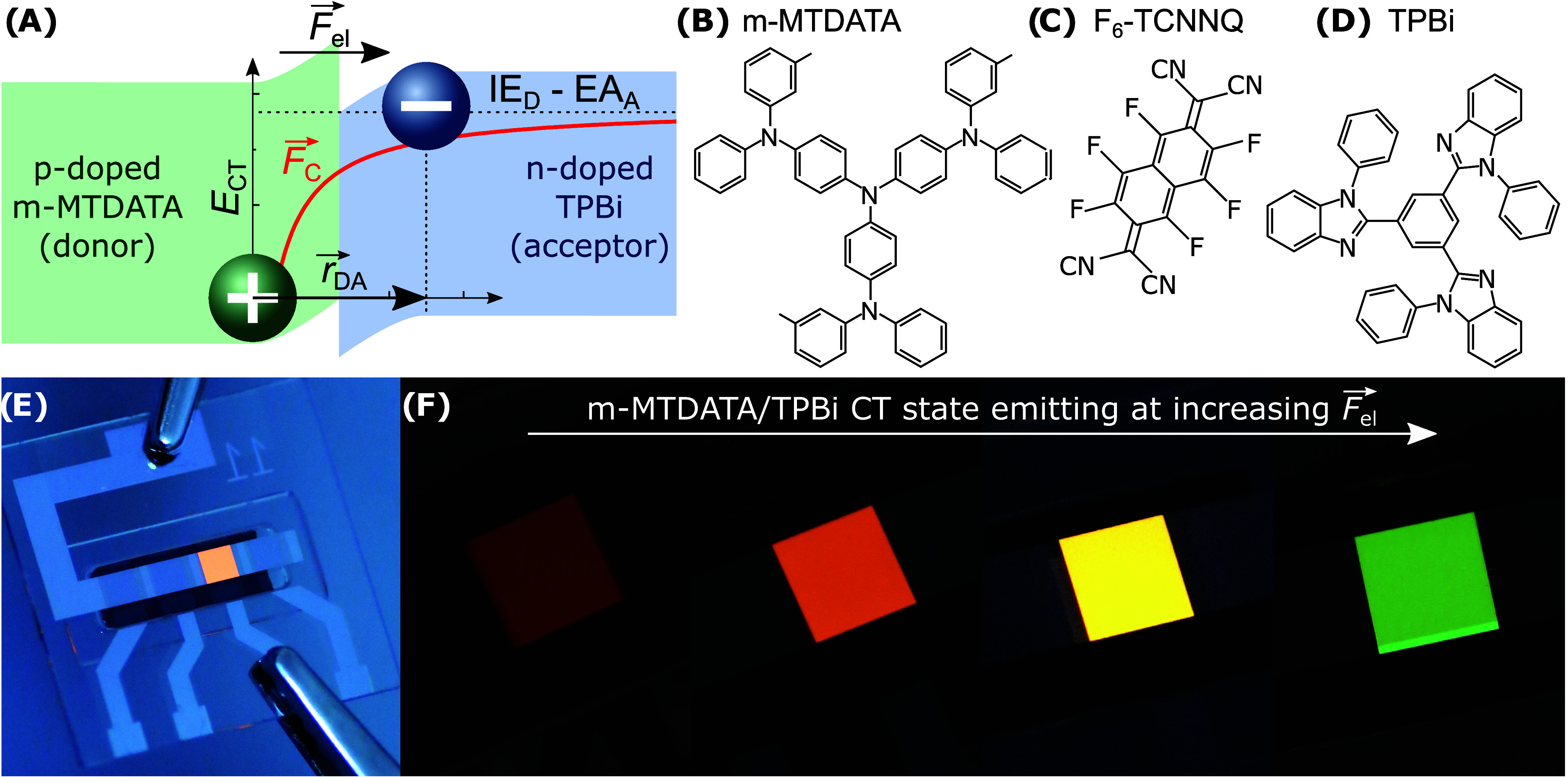
(A) Sketch of several parameters contributing to the CT-state energy *E*_CT_, cf. eq 1. Molecular structures of (B) the donor material m-MTDATA, (C) the *p*-dopant F_6_-TCNNQ, and (D) the acceptor molecule TPBi. Photographs of (E) the general device layout, see the Experimental Section for fabrication details, and (F) the shift of the emission color of the m-MTDATA/TPBi CT state with increasing interface electric fields.

The CT-state energy *E*_CT_ is influenced by several parameters, such as frontier orbital energies or the Coulomb binding energy, exemplified in Figure 1A. As introduced by Vandewal,^1^ it can be written as
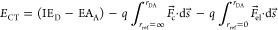
1
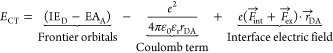
2

Note that this estimation omits polarizability influences, as we only investigate relative energy changes within this study. The first term on the right-hand side of eqs 1 and 2 describes the involved frontier orbitals at the interface, i.e., the difference in ionization energy (IE) of D and the electron affinity (EA) of A, cf. Figure 1A. This accounts for the amount of work required to move an electron from the highest occupied molecular orbital (HOMO) of D to the lowest unoccupied molecular orbital (LUMO) of A when D and A are at infinite distance.^18^ So, the biggest handle for tuning the CT-state energy is the choice of materials.^19^

In a working device, D and A are in contact. The impact on *E*_CT_ due to electric fields that can appear at the interface is considered by the two remaining terms, omitting quadrupole and higher multipole orders. If the hole on a D molecule is considered to reside at *r* = 0, cf. Figure 1A, the change in electrostatic potential energy of an electron with charge *q* = −*e* moved from a reference position *r*_ref_ to the position of the CT electron–hole separation *r*_DA_ in the presence of an electric field *F* is given by the negative of the work done by the electrostatic force, e.g., Greiner,^20^ with *r*_ref_ being the position of zero electrical potential energy. In the second term of eq 1, the electric field *F⃗*_C_ is caused by the Coulomb interaction, with ε_r_ being the relative permittivity, ε_0_ the dielectric constant, and d*s⃗* an infinitesimal displacement element. This integral gives the well-known 1/*r* dependency shown in eq 2 and can be solved in one dimension since the vectors *F⃗*_C_ and *r⃗*_DA_ are aligned. Compared to an LE, a CT exciton has an increased electron–hole separation distance *r*_DA_. Hence, its singlet–triplet splitting and Coulomb interactions are smaller. The latter is often referred to as the binding energy and ranges typically below 0.5 eV for intermolecular CT excitons.^21^

The electron–hole pair of a CT state can be treated as an electric dipole with an extension 1 nm < *r*_DA_ < 5 nm. This measure is motivated by the distance between adjacent D and A molecules at the interface. The potential energy of the electric CT-dipole moment *p⃗*_CT_ = −*e*·*r⃗*_DA_ depends on the interface electric field *F⃗*_el_ which is considered by the third term in eq 1 and is added compared to ref (1). This interface electric field *F⃗*_el_ = *F⃗*_int_ + *F⃗*_ex_ comprises the contributions of the energy level bending at the interface, referred to as intrinsic electric field *F⃗*_int_, and the extrinsic electric field *F⃗*_ex_ that can be induced by an applied external voltage. Considering the classical treatment of semiconductor p–n junctions as in pertinent textbooks and canonic coordinates (from D to A is the positive spatial direction *x*), the magnitude *F*_int_ has negative values, while a forward external bias induces a positive value for *F*_ex_, e.g., Sze and Kwok.^22^ If we assume that *r*_DA_ is smaller than the depletion region of the p–n junction, one can approximate the electric field to be constant over *r*_DA_ and the third term simplifies according to eq 2. This assumption is reasonable for our material system (cf. SI Section S11 for an estimation of the depletion width) but not strictly correct, i.e., the electric field does actually vary slightly over the extension of the CT exciton. We introduce this approximation, however, only for simplified estimation purposes. Finally, it is important to point out that the direction of the CT dipole *p⃗*_CT_ matters. Electric fields can only influence the electric dipole if their vectors are not orthogonal to each other. In this manuscript, as indicated in the TOC figure, we use a system where the electric fields and the dipole moments *p⃗*_CT_ are designed to be orthogonal to the interface plane, hence simplifying the scalar product in eq 2 to a one-dimensional multiplication.

As CT states are omnipresent at D–A type interfaces, OPV research has pointed out the influence of energy level bending on CT-state energies and the implications on exciton splitting.^23^ Previous studies have reported a blue shift of the CT emission color with increasing applied bias for planar, abrupt D–A junctions.^24−27^ Zhao, Song et al. suggested an enhanced contribution of LE emission from the bulk material with increasing bias.^24^ Al Attar and Monkman suspected a decreased equilibrium electron–hole separation *r*_DA_ for increased biases.^25^ Hörmann and Neher et al. discussed both the possibility of electric field influences and state filling using a hybrid organic–inorganic D–A system.^26,27^

This study builds on these preliminary ideas and presents a model system that marks a new approach to test the hypothesis of field influences. We gather extensive experimental evidence on how *E*_CT_ can be tuned both by *F*_int_ and *F*_ex_, cf. the third term in eq 2, combining techniques from OLED and OPV research. We use a dedicated intermolecular D–A system consisting of two doped organic layers on top of each other, forming a planar organic p–n heterojunction. This simple design produces an ensemble of CT dipoles *p⃗*_CT_ directed orthogonal to the interface plane and allows us to investigate both electric field contributions separately:

(I) *F*_int_ is adjusted by inserting intrinsic (undoped) bulk-material layers on both sides of the D–A interface (forming a p-i-n junction). By increasing the intrinsic layer thickness, we can adjust *F*_int_ at the planar interface.
(II) *F*_ex_ is tuned by applying external voltages. As we use doped layers, a significant share of this voltage drops over the interface at low forward bias. At high forward bias, the interface becomes very conductive (as motivated by the Shockley equation) and other resistance contributions like space-charge limitations or nonohmic injection prevail.

Exploring these two scenarios, we measure the emission color of the CT state and its absorption characteristics. Using a single D–A system of [m-4,4′,4″-tris(N-3-methylphenyl*N*-phenyl-amino)triphenylamine] (m-MTDATA) and [2,2′,2″-(1,3,5-benzenetriyl)-tris(1-phenyl-1-H-benzimidazole] (TPBi), see the molecular structures in Figure 1B–1D, the CT emission color can be tuned significantly from red over orange and yellow to green, cf. Figure 1F, while the LE emission does not contribute.

An assessment of the investigated D–A interfaces using classical semiconductor physics shows that the expected electric field variations can explain the measured spectral shifts. This finding supports the hypothesis of *E*_CT_ being field-dependent. While *E*_CT_ is often referred to as a fixed quantity,^25,28^ we want to emphasize that this understanding can be misleading, especially for planar systems with a high degree of interface CT dipole alignment. In bulk heterojunctions (BHJ), by contrast, where donor and acceptor molecules are blended in a single layer, the angle between *p⃗*_CT_ and *F⃗*_ex_ is random. Using such a system for comparison, we cannot identify a significant emission shift (see SI, Section S13), as was already evidenced by previous studies.^25,29^

## Conception

### Stack Design

To study *E*_CT_ as a function of the internal and external electric fields, *E*_CT_ = *E*_CT_ (*F*_el_ = *F*_int_ + *F*_ex_), we use a type-II organic planar heterojunction (PHJ), comparable to refs (25,30). This conception generates an ensemble of *p⃗*_CT_ directed orthogonal to the interface plane, as recently evidenced for a material system comparable to ours.^29^ In this study, we use thin evaporated films of m-MTDATA and TPBi, as their interface was reported to form an efficient emissive CT state.^31,32^ These films are sandwiched between aluminum and indium tin oxide (ITO) electrodes.

**Figure 2:**
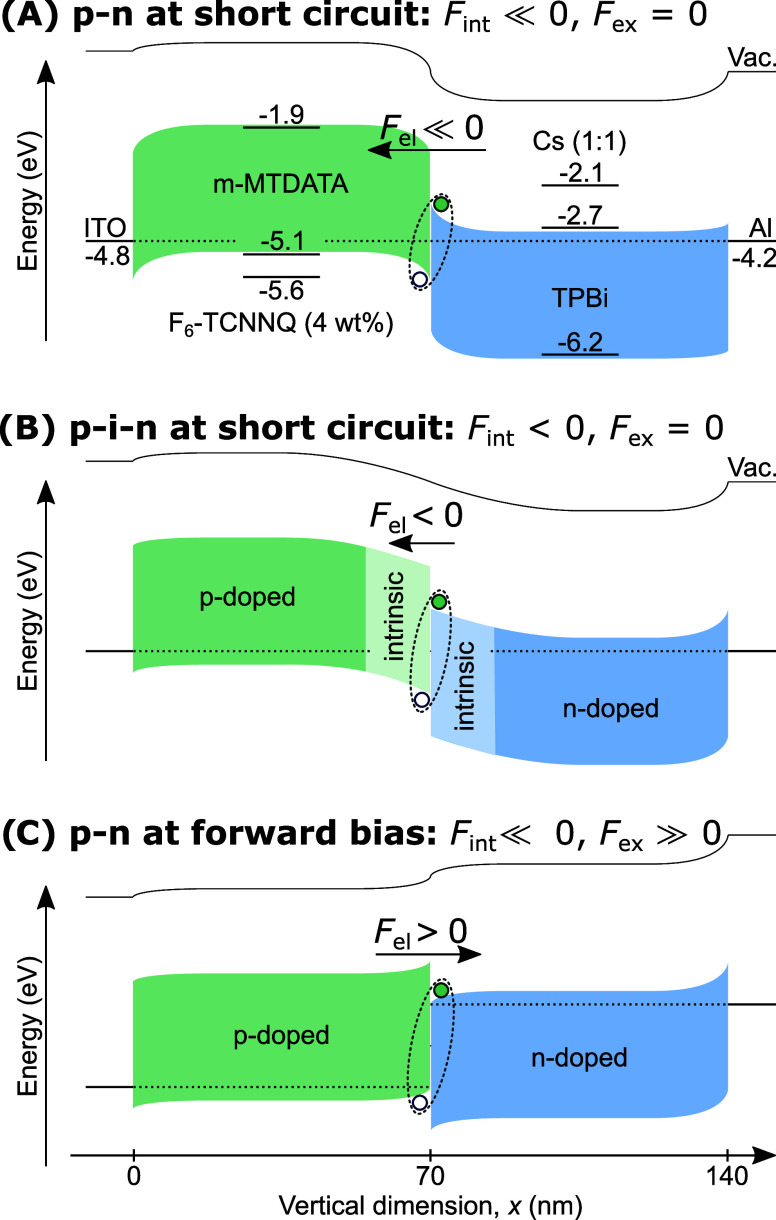
Sketch of the energy level diagrams, materials, CT excitons, and interface electric field magnitudes *F*_el_ = *F*_int_ + *F*_ex_ for different experimental conditions: (A) abrupt p–n junction at short-circuit (SC) condition, (B) p-i-n junction with intrinsic layers reducing *F*_int_ at the interface, (C) abrupt p–n junction under forward bias causing positive *F*_ex_ and a positive total electric field *F*_el_. Energy values are taken from refs (33−35). The dashed lines indicate the (quasi) Fermi levels.

Figure 2 presents the electron potential energy for several experimental scenarios realized in this study. It also indicates the used materials and their respective HOMO and LUMO levels, the energy levels of the neutral dopants, the CT excitons, the energetic separation of electron and hole indicated by ellipses, and it sketches the (quasi) Fermi levels and electric fields in the stack. The magnitude of the total electric field at the interface *F*_el_ (*x* = 70 nm) is proportional to the derivative of the vacuum energy level *E*_Vac_ at *x* = 70 nm, *eF*_el_ = d*E*_Vac_/d*x*. So, the vacuum level bending at the interface gives a direct measure of the encountered electric interface field, which we intentionally tune by the introduction of intrinsic layers or the application of an external voltage.

In contrast to the study of Al Attar and Monkman,^25^ we use doped organic films as shown in Figure 2A, forming an organic p–n heterojunction. Here, m-MTDATA is doped with 4 wt % of F_6_-TCNNQ [2,2′-(perfluoronaphthalene-2,6-diylidene)dimalononitrile] and TPBi is doped with Cesium (1:1). Estimation of the respective doping concentrations is found in SI Section S11. Note that we use the term p–n junction from a conceptual point of view. The current density–voltage (*J*–*V*) curve of the abrupt p–n configuration does not imply a significant rectification behavior but is governed by high leakage currents, cf. Figure S1. These leakage currents can arise from the strong energy level bending at the interface causing heavy charge-carrier tunneling and potentially dopant-mitigated charge-carrier passage through the interface energy barrier.^36−38^ As can be seen in Figure S1, the introduction of intrinsic layers significantly reduces leakage currents and boosts the electroluminescent external quantum efficiency (EQE_EL_) by 3 orders of magnitude.

Compared to the previously investigated undoped D–A systems, the doped layers introduced in this work provide two significant advantages: First, the built-in potential between the two organic layers is significantly enhanced. This leads to a high (negative) *F*_int_ at the interface, in the order of 300 mV/nm (see calculation below). By introducing intrinsic layers of increasing thickness at the interface (p-i-n junction, cf. Figure 2B), which we can finetune on the nanometer scale because we use thermal evaporation for film deposition, the absolute value of *F*_int_ at the interface can be deliberately reduced. The intrinsic layers are fabricated symmetrically, that is, a 4 nm intrinsic layer thickness implies 2 nm of undoped m-MTDATA and 2 nm of undoped TPBi forming the D–A interface, while the thickness of both doped layers is reduced to 68 nm each. Thus, the overall film thickness of 140 nm and the thickness of the D and A layers (70 nm each, cf. Figure 2B) are kept constant to exclude spectral shifts due to a changing optical cavity effect. Second, doped layers ensure both a low injection and transport resistance at reasonable driving conditions as investigated in a recent study from our lab.^39^ As a result, much of the applied bias can be expected to drop over the D–A interface at low forward bias, producing a significant extrinsic electric field *F*_ex_ (cf. Figure 2C). At high forward bias, the interface becomes highly conductive and other resistance contributions like space-charge limitations or nonohmic injection (here potentially from the cathode) are expected to restrict the device current.

### Estimation of Interface Electric Fields

In this section, we estimate the influence of both electric field contributions *F*_el_ = *F*_int_ + *F*_ex_ at the D–A interface of our samples. The outcome should not be understood as an exact result but rather as a ballpark figure telling us what experimental variations to expect. Note that *F*_int_ is negative for the canonic spatial coordinate *x* (from D to A is the positive spatial direction as shown in Figure 2), while *F*_ex_ is positive under forward bias. Both vectors are assumed to be orthogonal to the interface plane, which is why we only consider their magnitudes.

**Figure 3:**
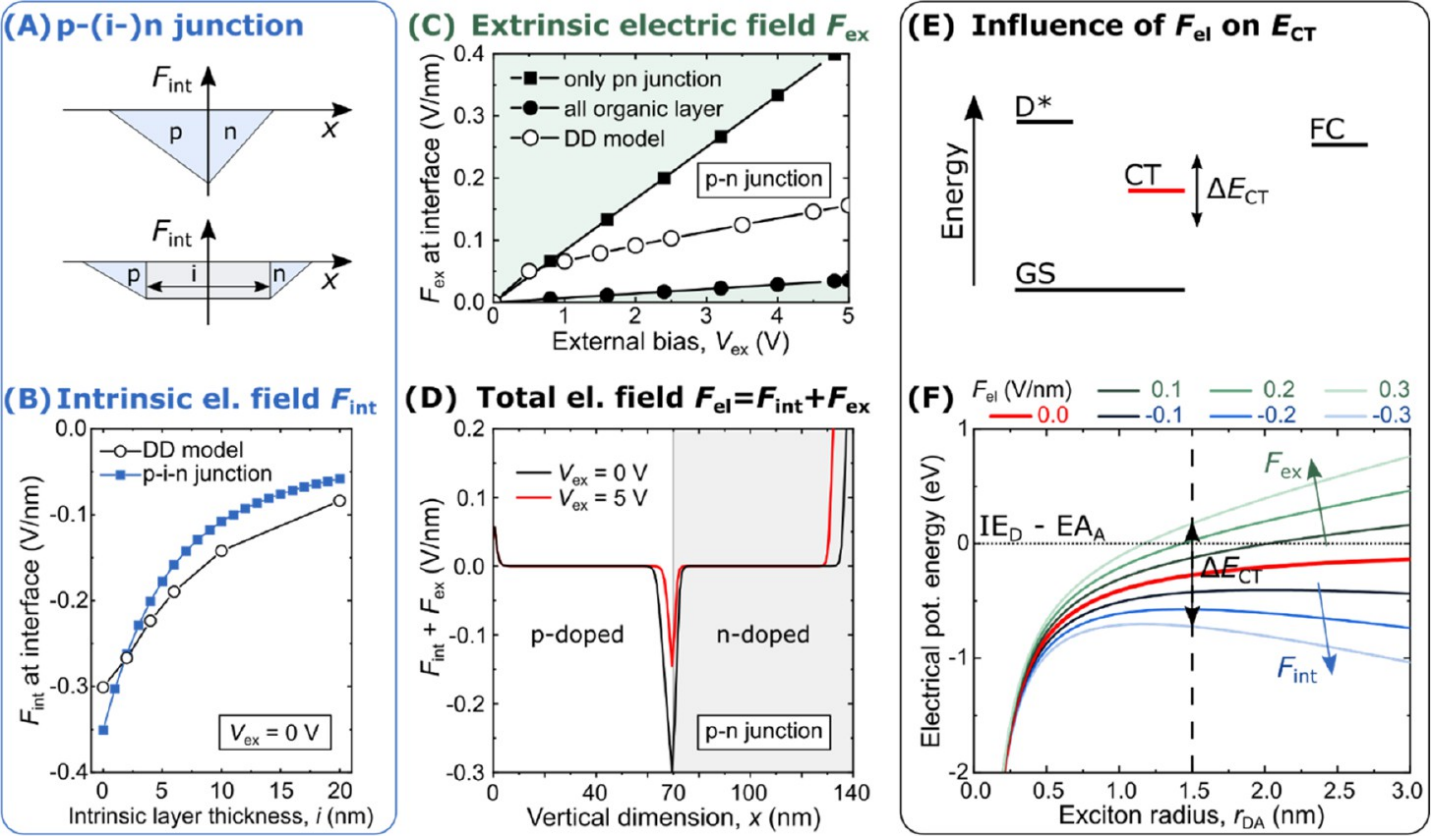
Estimation of electric interface fields: (A) Classical understanding of *F*_int_ at a semiconductor p-(i-)n junction following ref (22). (B) Dependence of *F*_int_ on the intrinsic layer thickness using the classical p-i-n junction approach and a drift-diffusion (DD) model. (C) *F*_ex_ at the D–A interface (*x* = 70 nm in D) as a function of the external bias for the p–n junction (*i* = 0 nm). (D) Total electric field distribution in the p–n stack (*i* = 0 nm) under varying external bias, according to the DD model. (E) Shift of *E*_CT_ relative to the donor singlet (D*), free charge (FC) level, and ground state (GS) with changing interface electric field. (F) Rationalization of the *E*_CT_ shift by eqn 2, where the pure Coulomb potential (red) is superimposed by *F*_el_ = *F*_int_ + *F*_ex_, which shifts *E*_CT_ by several 100 meV for typical CT exciton radii in our devices.

The intrinsic electric field *F*_int_ at the p-(i-)n junction can be calculated following classical semiconductor physics, as presented in ref (22), cf. Figure 3A. This estimation considers merely a one-electron picture and is suited for low current densities when electron–electron interactions are not dominant and the assumption of a depleted space-charge region is valid. With the material parameters and experimentally accessible doping concentrations at hand, *F*_int_ at the interface of the p-(i-)n junction can be calculated as a function of the intrinsic layer thickness. The results are shown in Figure 3B labeled “p-i-n junction”. See SI Section S11 for the detailed calculation. A second approach to estimate *F*_int_ is a drift-diffusion (DD) model. Here, we utilize the commercial software Setfos to calculate the electric field at the interface, using the same parameters as above. See SI Section S12 for specifications on the DD model. Both approaches result in similar characteristics, as presented in Figure 3B. They suggest that we can expect a shift Δ*F*_int_ ≈ 200 mV/nm when introducing 20 nm of intrinsic organic layer at the interface, i.e., 10 nm on each side of the junction.

The assessment of *F*_ex_ due to an applied external voltage *V*_ex_ is much more complex. We tried measuring the illumination-dependent short-circuit density as a function of the open-circuit voltage, *J*_SC_(*V*_OC_), as presented in refs (27,40), to exclude the series resistances in the devices. But we failed due to their high leakage currents. So, we estimate the following: If we assume the external voltage to drop evenly across the entire organic film of 140 nm, we could expect a change of 36 mV/nm at *V*_ex_ = 5 V, as indicated by the solid black circles in Figure 3C. On the other extreme, we assume the voltage to drop merely across the depletion region of the p–n junction, which is around 12 nm thick at *V*_ex_ = 0 V, see SI Section S11 for the estimation details. Here, one could expect *F*_ex_ to reach about 400 mV/nm at *V*_ex_ = 5 V, as indicated by the solid black squares in Figure 3C. Both estimates are oversimplified and only provide boundaries. Thus, the two green-shaded areas in Figure 3C define the expectation limits. The DD model solved in Setfos provides a more rational estimate within these boundaries. It suggests that for the fully doped p–n junction *F*_ex_ shifts by more than 100 mV/nm when applying *V*_ex_ = 5 V, cf. Figure 3C,3D.

Combining these two estimations, Figure 3E,3F presents how we can understand the dependency *E*_CT_ = *E*_CT_(*F*_int_ + *F*_ex_). Figure 3F considers the hole to reside at *r* = 0. The red line represents the Coulomb attraction of the electron–hole pair, lowering the original energy *E*_CT_ = IE_D_ – EA_A_ depending on their separation *r*_DA_. As introduced, *F*_int_ is negative and therefore lowers *E*_CT_, cf. eq 2. By contrast, *F*_ex_ is positive at forward bias and increases *E*_CT_. If we consider the above-estimated shifts Δ*F*_el_ = Δ*F*_int_ + Δ*F*_ex_ ≈ 200 + 100 mV/nm and a typical *r*_DA_ in the range of one to a few nanometers, our assessment yields a change of *E*_CT_ of several 100 meV. This impacts the relative position of *E*_CT_ to the donor singlet LE (D*) and the free charge (FC) levels, as indicated in Figure 3E.

In this picture, we assume *F*_el_ to be constant over the extension of the CT exciton. This simplification is only reasonable for low *V*_ex_, where *r*_DA_ is significantly smaller than the depletion width. Also, we consider the charge carriers of the CT states to be pinned at the interface at a fixed distance *r*_DA_. In Figure 3F, an example value of *r*_DA_ = 1.5 nm is used which would roughly correspond to the molecular separation. It means that we assume a step potential wall caused by the bulk materials that the charge carriers cannot overcome, keeping them at a fixed *r*_DA_, cf. Figure 2. This is another simplification, which would be wrong in light of ref (25). We want to point out, however, that a change in *r*_DA_ is not necessary to explain the observed emission shifts, though it probably still contributes. We address this further in the Discussion section. Finally, our assessment assumes a constant ε_r_ and thus a Coulomb interaction (second term in eq 2) which remains unchanged throughout the described scenarios. A transition from a p-i-n junction to a p–n junction, however, probably induces an increase of ε_r_ at the interface.^41,42^ This would translate into a reduced Coulomb interaction and an increase of *E*_CT_ in the case of an abrupt p–n junction. As we experimentally observe the contrary trend, cf. the following section, the change in ε_r_ may be present but is less significant than the field-induced energy tuning.

## Results

### Variation of the Intrinsic Layer Thickness

**Figure 4:**
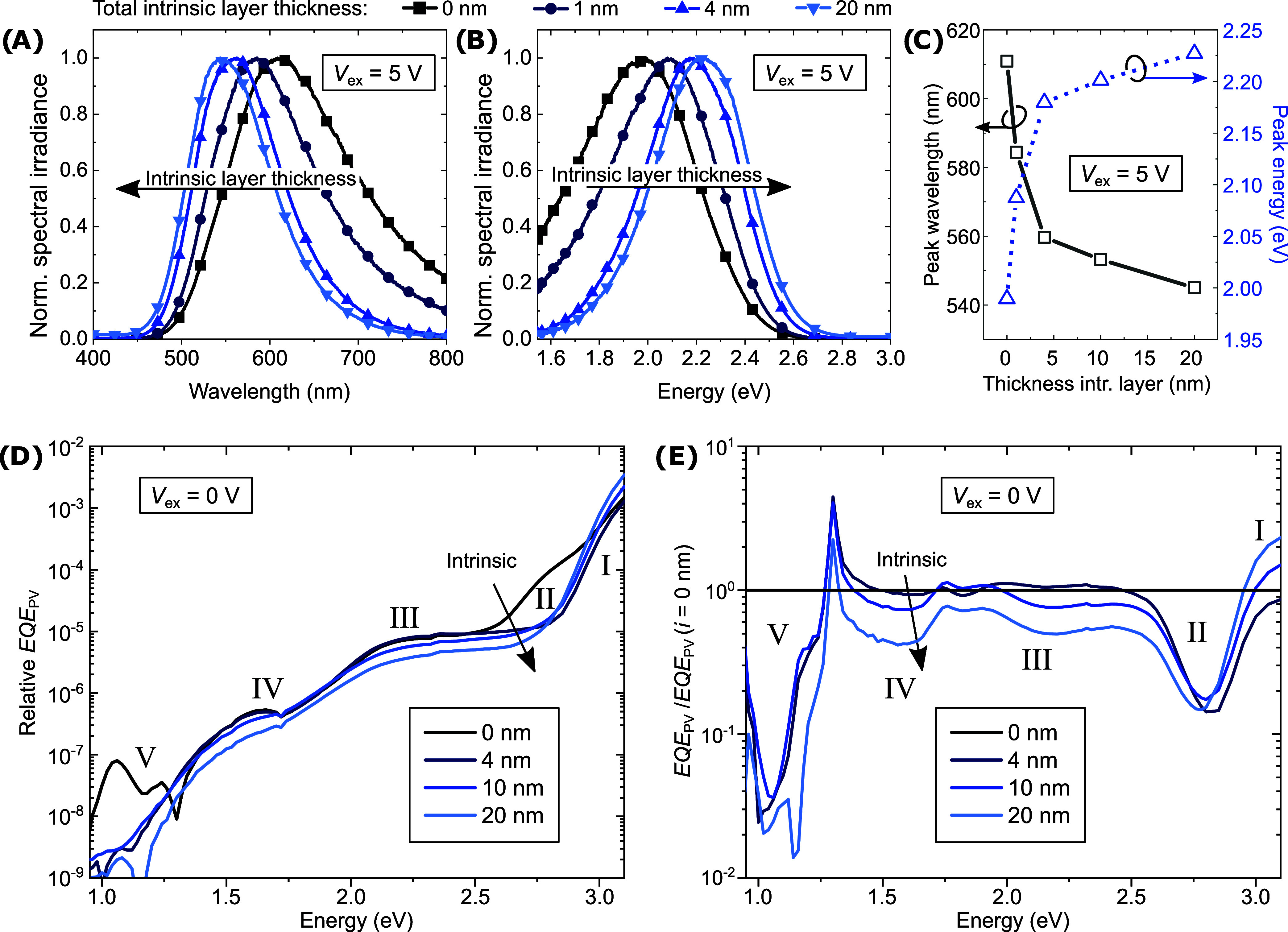
Experimental data for tuning the intrinsic layer thickness and thus *F*_int_: EL spectra over (A) wavelength and (B) energy at *V*_ex_ = 5 V normalized to the maximum intensity of each spectrum. (C) Emission peak shift obtained by Gaussian fits of the emission spectra at *V*_ex_ = 5 V. (D) sEQE_PV_ characteristics and (E) the relative changes to the abrupt p–n junction under increasing intrinsic layer thickness at *V*_ex_ = 0 V.

We now turn to the experimental assessment of whether *E*_CT_ can, according to our prediction, be tuned by a couple of 100 meV. First, we probe samples with increasing intrinsic layer thickness to investigate the influence of *F*_int_ while keeping the external bias constant at *V*_ex_ = 5 V. Figure 4A,4B show their normalized EL spectra versus wavelength and energy, respectively. The data are recorded over wavelength and converted to energy scale by Jacobian transformation.^43^ The emission peak is obtained by a Gaussian fit of the high-energy (low-wavelength) flank for both unit systems and presented in Figure 4C. The measured values of the EL peak are similar to the expected *E*_CT_ around 2 eV, corresponding to the HOMO–LUMO offset of the bulk materials reduced by their Coulomb interaction. No significant m-MTDATA bulk emission around 420 nm (2.9 eV)^31,32^ is observed. Increasing the intrinsic layer thickness from 0 to 20 nm increases (decreases) the peak-emission energy (wavelength) by over 200 meV (60 nm), approaching the emission wavelengths reported for the undoped systems, that is 520 nm for the bulk and 550 nm for the planar heterojunction.^31,32^ As presented in SI Section S13, we measure a peak emission wavelength of about 560 nm for the same D–A configuration in a bulk heterojunction configuration. The trend and magnitude of the relative energy change are in good agreement with the estimation of Δ*F*_int_ according to Figure 3B. The observed trend is a true spectral shift rather than a relative increase of the bulk LE emission, which does not contribute. The unnormalized spectra can be assessed in the SI Figure S2.

The energetic behavior of the CT state can also be probed by absorption spectroscopy. Figure 4D presents photovoltaic ultrasensitive external quantum efficiency (sEQE_PV_) characteristics at short-circuit conditions (*V*_ex_ = 0 V). It is important to note that this technique probes the combined ability of the sample to absorb photons, split excitons, and extract charge carriers because the photocurrent is measured. To better analyze the changes, the original data from Figure 4D are normalized to the signal of the abrupt p–n junction (no intrinsic layer) in Figure 4E. The spectra can be divided into five main regions, whose origins can be partially identified by further spectroscopic experiments, cf. SI Sections S6 and S7 and by investigating the sEQE of a bulk heterojunction (BHJ) device, cf. SI Section S13.

Region (I), the bulk absorption above 3 eV, corresponds to m-MTDATA,^31^ as TPBi absorbs above 3.5 eV, cf. Figure S6. Region (II), the shoulder around 2.8 eV, is where we expect the m-MTDATA/TPBi CT-state absorption feature. As indicated in a previous study from our lab with comparable materials, the peak absorption should appear several hundred meV above *E*_CT_, while the measured peak emission (here, somewhat above 2 eV, cf. Figure 4C) appears several hundred meV below *E*_CT_, indicating a significant Stokes shift.^44^ Thus, the absorption feature can be expected 600–800 meV above the emission peak. Since we neglected polarization effects in eqs 1 and 2, the absolute estimation of *E*_CT_ is vague. The feature in region (II) is only significant for the abrupt p–n junction in Figure 4D. When introducing intrinsic layers, it seems to blue-shift or decrease. For further indications, we compare the PHJ device with *i* = 20 nm with a BHJ device featuring 20 nm of mixed intrinsic layers, cf. SI Figure S17E. For the BHJ with a much higher CT-state absorption cross section, the shoulder in region (II) is significantly more pronounced. This further indicates that this feature originates from the m-MTDATA/TPBi CT state. As its absorption cross section in a planar device is very limited, however, we suspect that the CT signal is overlaid with the F_6_-TCNNQ characteristics, cf. SI Figure S6A–F. Region (III) seems governed by the F_6_-TCNNQ absorption, cf. SI Section S7, and the cationic m-MTDATA absorption, cf. SI Figure S6F. So, the change in the sEQE_PV_ signal with increasing intrinsic layer thickness in region (III) could correspond to a decrease in F_6_-TCNNQ contribution, as it is moved further from the interface. This behavior coincides with an increase in the bulk signal in region (I). Region (IV), the hump around 1.6 eV that seems to shift or decrease slightly with increasing intrinsic layer thickness, cannot be assigned to any particular absorption process via the conducted experiments, cf. SI Sections S6 and S7. Neither any of the bulk materials, their blend, nor the dopants have a pronounced absorption here. Region (V), the double peak above 1.0 eV, is only observed for the abrupt p–n junction. This feature corresponds to the F_6_-TCNNQ anion radical absorption.^35,45^ We support this interpretation by the experiments presented in Sections S6 and S7 in the SI.

### Variation of the External Voltage

**Figure 5:**
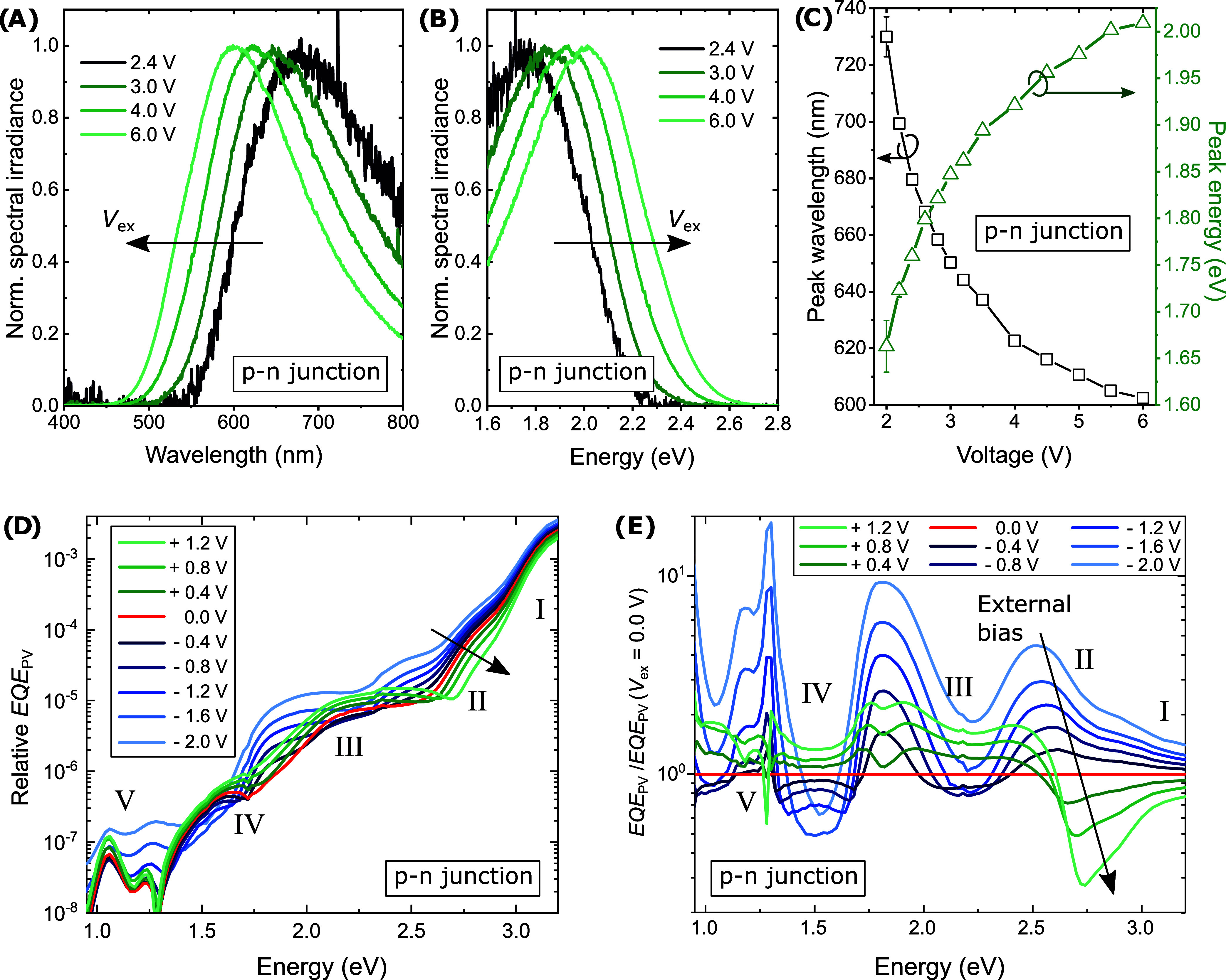
Tuning the external bias, i.e., *F*_ex_, in the abrupt p–n junction: EL over (A) wavelength and (B) energy at increasing *V*_ex_ normalized to the maximum intensity of each spectrum. (C) The corresponding shift of the emission peak as obtained by Gaussian fits. (D) sEQE_PV_ characteristics and (E) the relative change to short-circuit conditions.

Now, we exclusively study the impact of *F*_ex_. This is done using the abrupt p–n junction (fixed *F*_int_), sweeping the external bias *V*_ex_ and thus *F*_ex_. Figure 5A,B present the EL emission spectra under increasing forward bias over wavelength and energy, respectively. The emission was detectable with a spectrometer for *V*_ex_ > 2 V. Importantly, by increasing the applied bias from 2 to 6 V, the peak emission energy (wavelength) shifts by about 300 meV (120 nm), as presented in Figure 5C, corresponding to a color shift from red to yellow/green. We call attention to that the emission spectra again do not show a significant contribution from the m-MTDATA LE states, which would be expected around 420 nm (2.9 eV), cf. Photoluminescence (PL) measurements in Figure S3 or refs (31,32). This observation supports the notion of a shift of the emission energy rather than a gradual mixing of LE-state and CT-state emission. All unnormalized spectra are presented in SI Section S2. Comparing this behavior to our control device (the m-MTDATA/TPBi bulk heterojunction (BHJ) presented in SI Section S13), we can see that the BHJ configuration, by contrast, does not exhibit a significant spectral emission shift under increasing forward bias.

Figure 5D presents the *sEQE*_PV_ spectra under varying forward bias, and Figure 5E presents the same data normalized to the short-circuit condition *V*_ex_ = 0.0 V. Note that the applied bias, and therefore *F*_ex_, is different from the EL experiments. Yet, we can investigate potential trends and even apply negative bias. The peak positions in regions (I) and (V) are rather stable and only vary in magnitude. Here, as a general trend, a negative bias seems to enhance charge-carrier extraction, which is a common OPD behavior. Region (II) by contrast, where we expect the m-MTDATA/TPBi CT-state absorption, exhibits a lateral shift. The feature is blue-shifted with increasing forward bias and red-shifted under increasing reverse bias. This trend is clearly visible in Figure 5E. Regions (III) and (IV) in Figure 5D show a more complex behavior. Though the absorption features change under *F*_ex_ variation, it remains unclear to us whether we detect a change in the energetic configuration, the charge-extraction probability, the absorption coefficient, or a combination of all effects. As the features here probably originate from a mixture of several exciton absorption pathways, cf. SI Section S6, a clear interpretation of the measured trend does not seem feasible.

### Further Experiments on the Same D–A System

To further characterize the employed devices, we present their short-circuit current density (*J*_SC_) and open-circuit voltage (*V*_OC_), cf. Figure S5. Increasing the intrinsic layer thickness from 0 to 20 nm shifts the *V*_OC_ from about 1 V to beyond 2 V. This trend could be explained by an increase in *E*_CT_ or by a decrease of the nonradiative voltage losses (Δ*V*_nr_) with increasing intrinsic layer thickness.^46,47^ Based on our EQE_EL_ measurements we can estimate the nonradiative voltage losses Δ*V*_nr_, cf. Table S1.^48^ Note that the injection current for EL measurements is much higher than the photocurrent under 1 sun illumination, however, which induces an error in this estimation. By inserting intrinsic layers at the interface, Δ*V*_nr_ is reduced by 200 mV which coincides with the decrease in leakage current as observed from the OLED *JVL* characterization in Figure S1. However, the *V*_OC_ increase is beyond the interval we can expect from a combination of the electric field-induced effect (about 200 mV/nm, cf. Figure 3B) and nonradiative voltage losses (another 200 mV, cf. SI Table S1).

We also measure the effects of the intrinsic layer thickness on the EL onset of the p-(i-)n devices. Using a photomultiplier tube for the highest sensitivity, we could detect an increasing turn-on voltage with increasing intrinsic layer thickness, cf. SI Figure S4. This may be related to an increase in the optical gap and *E*_CT_. But it could also be explained by an increased series resistance caused by the thicker intrinsic layers.

To exclude the influences of *F*_ex_ on the intrinsic layer variation, i.e., to probe the CT emission without applying a bias, we conduct photoluminescence (PL) experiments. However, as our samples have a very high mismatch between bulk and CT absorption cross section, the CT-state PL contribution at *V*_ex_ = 0 V cannot be isolated from the m-MTDATA PL emission, see SI Figure S3.

### Further Material Combinations

To investigate the merit of our experimental concept on different material systems, we fabricate comparable devices from other organic materials. First, we aim to further reduce the impact of the injection and transport resistance and fabricate the same material combination with an increased doping concentration of 10 wt % on each side. Also, we replace Cs with ditungsten tetra(hpp) [W_2_(hpp)_4_] to better track the experimental doping concentration and prevent potential dopant diffusion. This combination yields even higher leakage currents and no light emission can be detected, cf. SI Figure S11. These results indicate a limitation to our stack design, as highly doped layers beyond a certain threshold suffer from a destructive level of leakage current.

Second, we investigate BF-DBP:F_6_-TCNNQ/B4PYMPM:W_2_(hpp)_4_ as a different D–A system. The full names and figures are found in SI Section S10. This host material combination has been studied as a bulk heterojunction in our lab, showing both efficient light-harvesting and light-emitting performance.^44,49^ Here, we again use doped layers and the same stack architecture as above, testing different doping concentrations of 2 and 4 wt % on each side of the junction, and varying the intrinsic layer thickness as previously introduced. While the undoped materials operated in a bulk heterojunction have yielded promising performance,^44^ the doped planar heterojunctions suffer from significant interface leakage, which prohibits the measurement of *V*_OC_, cf. SI Figure S14. EL emission can only be detected from samples with intrinsic layer thicknesses above 4 nm, which show a comparable EL shift as measured for the m-MTDATA/TPBi system, see Figure S12B. It thus seems that the archetypical, doped OLED materials m-MTDATA and TPBi represent a suitable system because of contained leakage currents, the comparably high radiative quantum yield, and the formation of electric dipoles that are well aligned to the external electric field.

## Discussion

We now discuss the main hypothesis that the interface electric field can significantly influence the CT-state energy *E*_CT_. The design of our study is inspired by two previous investigations employing planar organic heterojunctions^25,29^ and by the ideas put forward by Hörmann and Neher et al. studying hybrid planar interfaces.^26,27^ These studies encountered voltage-dependent blue shifts of the CT-state emission in planar heterojunctions, a phenomenon which we reproduce herein. To contribute to the understanding of these variations, we present organic p-(i-)n junctions as a new model system to study the underlying physics. This system holds the advantage that we can separately control intrinsic and extrinsic field variations, i.e., Δ*F*_int_ and Δ*F*_ex_. Moreover, the doped layers ensure that a large fraction of the applied voltage drops over the heterojunction under low forward bias. Thus, we can reach a much wider range of emission shifts than reported earlier, notably visible to the bare eye, cf. Figure 1F and SI video. A disadvantage of our device design is a high interface leakage current, which renders methods like *J*_SC_(*V*_OC_) difficult or impossible to conduct. That is why, we cannot exactly quantify Δ*F*_ex_ at the interface, as in ref (27), but have to estimate it by a drift-diffusion model.

In line with the presented theoretical estimations, the changes in Δ*F*_int_ and Δ*F*_ex_ agree qualitatively with the recorded significant changes in the peak-emission energy, cf. Figures 3, 4C and 5C. The measured *sEQE*_PV_ characteristics indicate similar trends but are less straightforward to interpret. Based on the estimation that the CT-state absorption should be located 600–800 meV above the emission energy^44^ and a complementary experiment using a BHJ configuration, cf. SI Section S13, we suspect the m-MTDATA/TPBi CT-state absorption is around 2.8 eV. With a reduction of the (negative) intrinsic electric field, the feature around 2.8 eV decreases or blue-shifts in Figure 4D. When applying an external voltage, the same feature can be shifted laterally, cf. Figure 5D. We have to stress, however, that the absorption cross section of the interface CT states in a planar heterojunction is small compared to the bulk and dopant materials. The impact of F_6_-TCNNQ on m-MTDATA is clearly visible, cf. Figure S6, inducing an absorption feature in the same spectral region. So, it is highly likely that the F_6_-TCNNQ absorption obscures the m-MTDATA/TPBi CT-state signal in our *sEQE*_PV_ data. It could be argued that these experiments do not undeniably prove our hypothesis, and this is why we discuss further explanation approaches below.

A comprehensive and well-assessed picture relating the static disorder of amorphous materials to *E*_CT_, its emission, and absorption characteristics was brought forward by Yan, Nelson, and co-workers.^47^ This study gives a plausible explanation as to why a filling of the CT-state manifold can be identified as an origin of blue-shifted EL spectra with increasing applied voltage or device current. Linderl, Brütting, and co-workers further discussed this shift for organic bulk heterojunctions focusing on a comparison between preferentially amorphous and crystalline donor materials.^50^ Also, in a study on hybrid tin oxide/organic heterojunctions, state filling and the corresponding quasi-Fermi level splitting (QFLS) at the interface have been proposed as the cause for EL shifts.^27^ In this scenario, the undetected shift of the CT-state absorption in our experiments could be explained: Under absorption conditions, the state-filling effect would be less pronounced and thus induce only minor spectral shifts.

Both explanation scenarios appear reasonable and could potentially be superimposed in D–A heterojunctions: The filling of the density of states (DOS) at the D–A interface and the corresponding increase in the mean recombination energy sounds very plausible. Likewise, the perception of an intermolecular CT state as an electric dipole that is subject to an interface electric field, as introduced herein, is straightforward from a classical electrostatic standpoint. In our study, we use the term extrinsic electric field which gives rise to a linear change in *E*_CT_ according to eq 2. In a first approximation, this induces a linear dependency between the voltage over the interface *V*_int_ and *E*_CT_. Interestingly, the study by Hörmann and Neher et al. also approximates a linear relation between QFLS and *V*_int_.^27^ This formal similarity raises the question about the physical differences and how to test their validity. While our data cannot completely disentangle this puzzle, our results can contribute with some meaningful insights.

In ref (27), a dependence of the EL peak energy on the interface doping concentration has been derived. Our data set can now deliberately test this scenario, which we refer to as a tuning of the intrinsic electric field *F*_int_, realized by the introduction of intrinsic layers at the D–A interface. Figure S2 shows the absolute spectral change of all investigated p-(i-)n samples with voltage. Even when driving the abrupt p–n device (intrinsic layer thickness = 0 nm) at the highest voltages investigated, it does not reach the emission color of the devices featuring an intrinsic layer, even when their emission is barely detectable and their CT DOS filling must thus be much smaller. That means that *E*_CT_ has to depend on the intrinsic layer thickness, no matter the amount of DOS filling. In our understanding, this could have also been encountered in ref (27). There, a variation of *E*_off_ can be seen, which describes the energy offset between the valence Fermi level and the hole peak population. This offset energy *E*_off_ equals the doping-dependent energy level bending at the interface. At extremely heavy doping, *E*_off_ could theoretically approach zero which corresponds to a maximum energy level bending and the alignment of IE_D_ and EA_A_ to the Fermi level at short-circuit conditions, cf. Figure 2. In other words, the Fermi level would correspond to the acceptor HOMO and donor LUMO levels, and the depletion regions would be extremely thin. At experimentally reasonable or no doping, e.g., below 10 wt %, *E*_off_ increases which corresponds to a reduced energy level bending (increased depletion width) and is described in our perspective by a decrease in the absolute value of *F*_int_ raising in turn *E*_CT_, cf. Figure 3B.

Our experiments thus provide a strong argument for the influence of *F*_int_ on *E*_CT_. Conceptually, the influence of *F*_ex_ is no different. Further indications originate from two literature references that compare the voltage-dependent emission shift of bulk and planar heterojunctions.^25,29^ The authors found that a planar junction comprises directed electric dipoles and exhibits significant wavelength shifts, while the bulk junction shows no peak shift. We can reproduce this result, cf. SI Section S13. This assessment aligns with our hypothesis that only an ensemble of directed dipoles aligned to the electric field, cf. third term in eq 2, should be altered by Δ*F*_ex_. For a random orientation of the interface dipoles, as encountered in a bulk heterojunction, the scalar product in eq 2 would take random angles and no net shift can be produced.

When driving the p–n device beyond 6 V, we encounter significant Joule self-heating, runaway currents, and eventually a quick device destruction.^51,52^ Even when operating the sample in a pulsed mode, the voltage-dependent wavelength shift seems to level off. The reason for this may be ambiguous. The Setfos model indicates that the cathode injection resistance becomes dominant in the device at high external voltages and that *F*_ex_ does not increase much further at high *V*_ex_, cf. Figure 3D. Also, when *E*_CT_ is sufficiently raised it may couple to the LE triplet states,^32^ cf. Figure 3E. While this process is not visible in the emission spectrum, it will open a severe loss channel for the high-energy CT states. As brought forward in ref (25), a reduction of *r*_DA_ and hence the electric dipole moment with increasing forward bias is also a reasonable scenario. This would in turn reduce *p⃗*_CT_ and, thus, the magnitude of the energy change that the extrinsic electric field can cause. Also, at high forward bias, the depletion region becomes small, comparable to *r*_DA_, and thus our estimation in eq 2 of a constant *F*_el_ over the extension of the CT exciton fails.

To further test the validity of the state-filling hypothesis, we run sEQE_PV_ measurements under DC bias illumination, cf. Section S8. If the CT absorption would shift under photoexcitation, this would give support to this hypothesis. However, even when exposing the samples to intense illumination of about 6 suns, no clear trend can be observed.

As for the EL experiments, a constant driving current should be used as an independent experimental variable for further experiments. If the charge carriers can be assumed to predominantly decay as CT excitons at the interface, this could provide a handle to exactly measure the interface voltage by a *J*_SC_(*V*_OC_) experiment.^27,40^ For example, it would be more reasonable to compare our devices in Figure 4 at the same current rather than the same voltage. The aforementioned heavy leakage current, however, which depends on the intrinsic layer thickness, prohibits such measurements. Nevertheless, follow-up research should identify a device with lower leakage currents so that an assessment of the interface voltage via *J*_SC_(*V*_OC_) is possible. Together with a dedicated scan of intrinsic and extrinsic electric fields, this could shed more light on the question of to what extent state filling and the extrinsic electric field contribute to the observed emission shift.

## Conclusions

In this article, we systematically fabricate and study planar organic m-MTDATA/TPBi D–A p-(i-)n heterojunctions that exhibit an emissive intermolecular CT state across a type-II interface. By varying the intrinsic layer thicknesses and the external voltage, we realize a device architecture that allows us to controllably and separately tune the intrinsic and extrinsic electric fields at the interface, *F*_int_ and *F*_ex_, and to study their respective influence on the CT energy *E*_CT_. With this approach, we manage to induce changes to the EL peak emission energy of more than 0.5 eV, corresponding to a color change from deep red (720 nm) to green (550 nm) using a single D–A material combination. From the spectral shape, we can conclude that we indeed detect a CT emission shift and not a mixing of CT and LE states. We show that these experimental trends can be explained by the interface electric field variations, which we estimate based on classical semiconductor physics and a drift-diffusion model. When investigating the absorption energy of the CT state by means of an sEQE_PV_ measurement, we can identify field-induced shifts in agreement with our hypothesis. We suggest, however, that the CT-state feature is obscured by competing subgap absorption channels of the system, which compromise a clear interpretation. Our results underline the importance of the interface electric field on the function of organic heterojunctions and demonstrate how CT states can be susceptible to the device design and the operation conditions both in light-emitting and light-absorbing devices.

## Experimental Section

The techniques and materials used for complementary experiments in the Supporting Information (SI) are specified in the respective SI section.

### Device Fabrication

All devices are fabricated by thermal evaporation under high vacuum (Kurt J. Lesker Company, evaporation pressure < 1 × 10^–6^ mbar) on 2.5 cm × 2.5 cm glass substrates of thickness 1.1 mm with a structured 90 nm ITO anode (Thin Film Devices, Inc.). The substrates are cleaned in an ultrasonic bath using *N*-methyl-2-pyrrolidone (NMP), deionized water, and ethanol. The p-doped layer consists of m-4,4′,4″-tris(*N*-3-methylphenyl*N*-phenyl-amino)triphenylamine (m-MTDATA; Synthon, sublimation cleaned) doped with 2,2′-(perfluoronaphthalene-2,6-diylidene)dimalononitrile (F_6_-TCNNQ, Novaled AG, sublimation cleaned) at a ratio of 4 wt %. The n-doped layer consists of 2,2′,2’’-(1,3,5-Benzenetriyl)-tris(1-phenyl-1-H-benzimidazole) (TPBi, Gute Chemie ABCR, sublimation cleaned) doped with cesium (SEAS) at a molar ratio of about 1:1. The evaporation rates for the Cs doping are determined by an ETL conductivity test of a BPhen:Cs layer and may therefore deviate from a 1:1 molar ratio. The intrinsic layers are fabricated by the pure bulk materials (m-MTDATA and TPBi) on doped layers with reduced thickness, keeping the full stack of organic layers always at 140 nm. 80 nm of Aluminum (Chempur) is used as a top electrode (cathode) and as an anode reinforcement. The thickness and deposition rates are monitored using a quartz-crystal microbalance (QCM). To prohibit air and moisture contamination, the device stacks are encapsulated under a nitrogen atmosphere after fabrication. The encapsulation glass (Sodalime glass, AMGTECH Korea) comprises a small cavity above the pixels that prevents direct contact between sensitive materials and a moisture getter (Dynic Ltd., China). It is attached to the substrate using an epoxy resin (XNR5516Z-L and XNR5590, Nagase Europa GmbH). The pixel area equals the overlap of the bottom and top electrodes, which is 6.44 mm^2^.

### Electroluminescence Spectroscopy

EL and PL spectra of the forward sample emission are taken with a calibrated UV–vis spectrometer (CAS 140CTS, Instrument Systems). The devices are run by a source-measure unit (Keithley Instruments 2400) operated by the measurement software SweepMe! (sweep-me.net). The peak emission wavelength (energy) was obtained by fitting the emission spectrum over wavelength (energy) around the peak by a Gaussian in Origin. The standard deviation is given as error bars in Figure 5C. Spectral conversion from wavelength to energy scale was performed by Jacobian transformation.^43^

### Sensitive EQE_PV_ Spectroscopy

The sEQE measurements are performed in air using the light of a 250 W halogen lamp (OSRAM HLX 64657, Germany) which is chopped at 72 Hz. The monochromatic light output of a double monochromator (Quantum Design GmbH MSHD-300A, Germany) is focused onto the sample and its generated photocurrent is measured under short-circuit conditions or biases as indicated. After preamplifying the signal by a current–voltage preamplifier (Stanford Research Systems SR 570), it is forwarded to a lock-in amplifier (Stanford Research Systems SR830) and integrated with a time constant of 1 s. A calibrated Si photodiode (Thorlabs FDS100-CAL) and InGaAs photodiode (Hamamatsu Photonics G12183_020 K, Japan) are used for determining the incident photon flux and subsequently, the *EQE* is calculated by dividing the photocurrent of the investigated device by it. The setup is controlled by the measurement software SweepMe! (sweep-me.net).

## Data Availability

All relevant experimental data, photographs, a movie, and the Setfos file are publically available here: 10.6084/m9.figshare.25887892

## Supporting Information Available

Supporting experimental data (Figures S1–S17) (PDF)
Abrupt p–n junction under varying forward bias (MP4)
