# Supplementary material for: Tuning Charge-Transfer States by Interface Electric Fields
Source: ACS Appl Mater Interfaces. 2024 Jun 6;16(24):31407–18. doi: 10.1021/acsami.4c04602 (PMC11194774; doi:10.1021/acsami.4c04602)
Supplement: Supplementary file 1 — am4c04602_si_001.pdf [file am4c04602_si_001.pdf]

Supporting information
Tuning charge-transfer states by interface electric fields

Anton Kirch*† [1,2], Jakob Wolansky† [1], Shayan Miri Aabi Soflaa[1], Stephanie Anna Buchholtz[1], Robert Werberger[1], Christina Kaiser[1], Axel Fischer[1], Karl Leo[1], Ludvig Edman[2], Johannes Benduhn[1], and Sebastian Reineke[1]*

[1] Dresden Integrated Center for Applied Physics and Photonic Materials (IAPP) and Institute of Applied Physics, Technische Universität Dresden, Nöthnitzer Straße 61, 01187 Dresden, Germany

[2] The Organic Photonics and Electronics Group, Department of Physics, Umeå University, Umeå SE-90187, Sweden

† These authors contributed equally to this work

*Correspondence: anton.kirch@umu.se

sebastian.reineke@tu-dresden.de

Contents

S1. OLED characterization (*JVL* and EQE_{EL}) 2
S2. Electroluminescence spectra 3
S3. Photoluminescence under external bias 4
S4. Electroluminescence turn-on behavior 5
S5. Photovoltaic characterization 6
S6. UV-vis absorption spectroscopy 8
S7. Further spectroscopy on F_6-TCNNQ 10
S8. $sEQE_{PV}$ measurements under illumination 12
S9. Asymmetric intrinsic layers 13
S10. Further material systems 14
A) m-MTDATA:F_6-TCNNQ (10 wt%) / TPBi: $W_2(hpp)_4$ (10 wt%) 14
B) BF-DBP:F_6-TCNNQ (2 wt%) / B4PYMPM:$W_2(hpp)_4$ (2 wt%) 15
S11. Calculation of depletion width, doping concentration, and intrinsic electric field 17
S12. Modeling of the p-n junction with Setfos 19
S13. Comparison to randomly oriented dipoles in a bulk heterojunction 21

S1. OLED characterization (*JVL* and EQE_{EL})

The samples investigated in the main manuscript are characterized by their light-emitting performance. The abrupt p-n junction (intrinsic = 0 nm) suffers from high leakage current [corresponding to an extremely low $EQE_{EL}(J)$], which decreases with increasing intrinsic layer thickness. All curves in Fig. S1 are measured from 0 V to 3 V and back to monitor Joule self-heating effects.

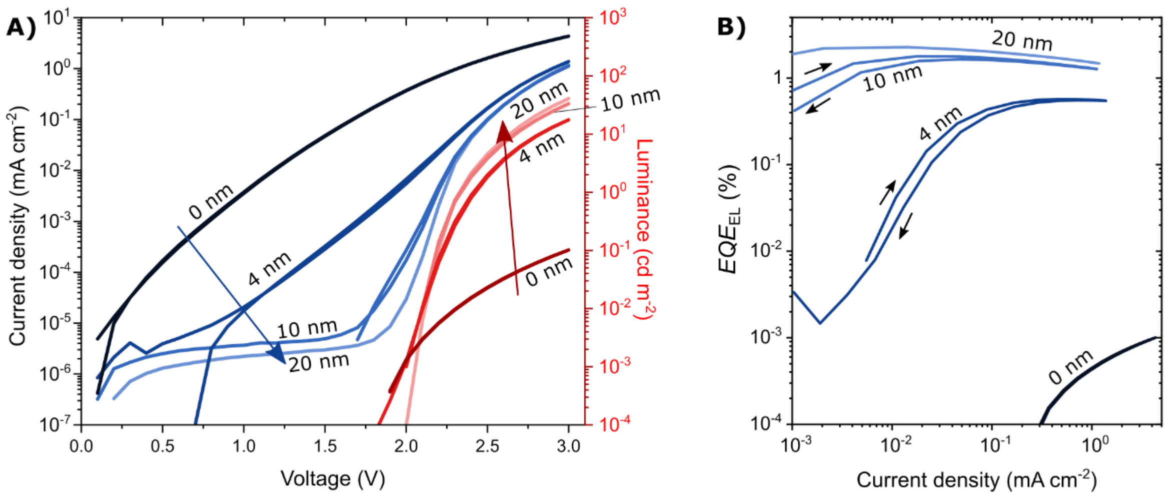

Figure S1. *OLED characteristics of the investigated p-(i-)n devices. A) Current density-voltage-luminance (JVL) characteristics depending on the intrinsic layer thickness and B) their respective EQE_{EL}. Each curve is measured forward and backward to monitor self-heating hysteresis.*

The EQE_{EL} is calculated with an EL spectrum taken at 0.5 mA (corresponding to 7.8 mA/cm^2) for every device. This induces a certain error in the EQE_{EL} calculation, as the emission spectrum changes with voltage. As we do not require exact EQE_{EL} values but rather want to compare the trend between the samples and determine the order of magnitude, this error source is not crucial.

The current-voltage values are taken by an SMU (Keithley Instruments 2450). The luminance is tracked with a calibrated silicon photodiode covering the full OLED pixel evaluated by a Picoamperemeter (Keithley Instruments 6485). The emission spectra are measured with a USB spectrometer (Ocean Optics USB4000) in a separate scan. All devices are operated and evaluated using SweepMe! (sweep-me.net).

S2. Electroluminescence spectra

The electroluminescence (EL) spectra of the samples investigated in the main manuscript are displayed depending on the applied forward voltage. The overall spectral shift between the samples relates to the intrinsic electric field variation at the interface with increasing intrinsic layer thickness. The thicker the intrinsic layer, the smaller the magnitude of the negative intrinsic electric field at the interface, cf. Fig. 3B in the main manuscript. This introduces an overall blueshift with increasing intrinsic layer thickness. Also, the voltage-induced spectral shift of individual samples becomes smaller with increasing intrinsic layer thickness. We account this to the fact that with increasing intrinsic layer the interface voltage drops over a widening depletion region and reduces the extrinsic electric field variation directly at the interface (across the CT-exciton dipole), cf. Fig 3A.

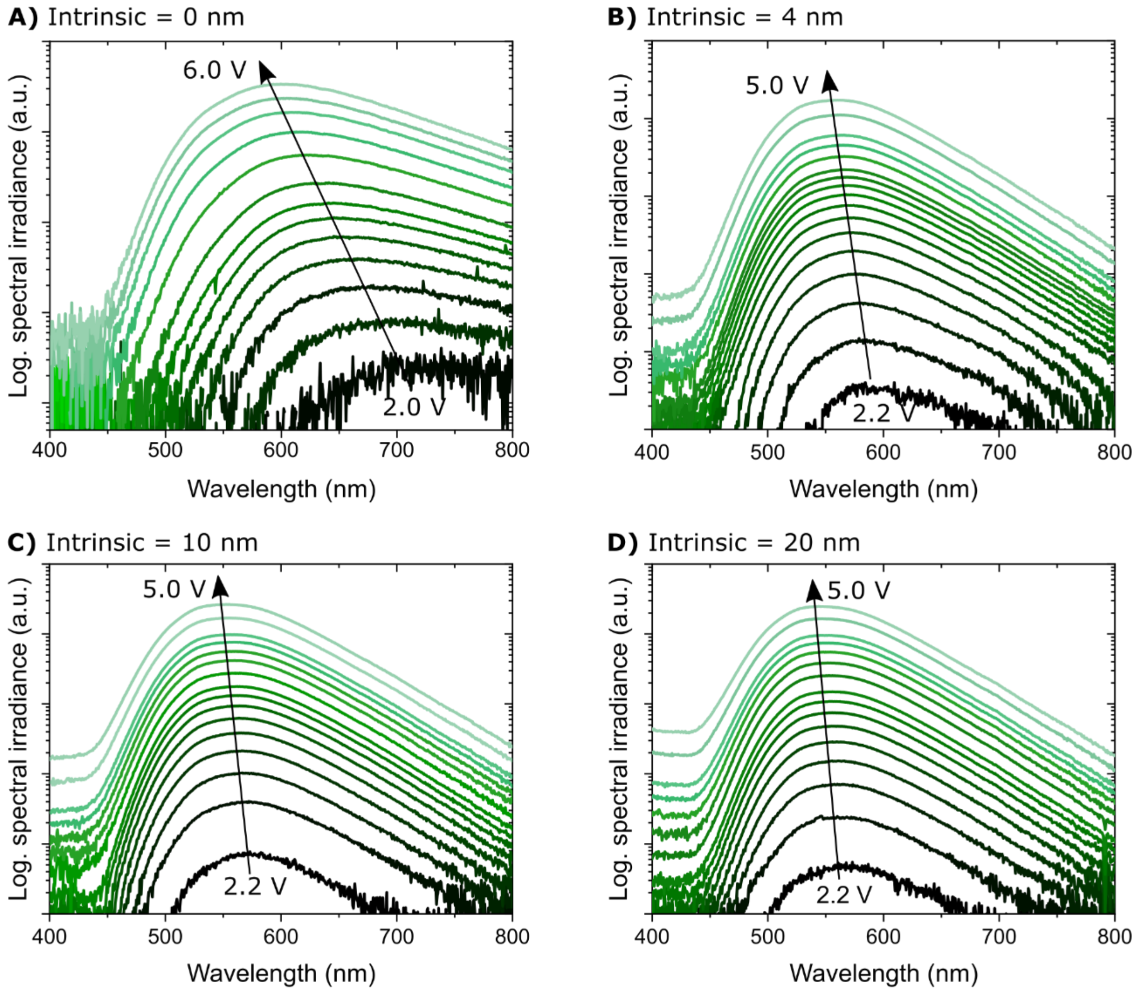

Figure S2. *Electroluminescence spectra under increasing forward bias of the investigated p-(i-)n samples for A) 0 nm, B) 4 nm, C) 10 nm, and D) 20 nm of intrinsic layer thicknesses.*

S3. Photoluminescence under external bias

The combined photoluminescence (PL) and EL of the p-(i-)n samples investigated in the main manuscript are recorded at different forward voltages under 365 nm illumination (UV LED Thorlabs M365L2 equipped with a corresponding band-pass filter Thorlabs FB370-10). The m-MTDATA emission at 415 nm is too bright to disclose the CT-state PL at V_{ex} = 0 V. At 365 nm, m-MTADATA absorbs mainly, cf. Fig. S6. The absorption cross-section of the interface CT state is too small to evoke a significant PL compared to the m-MTDATA emission. With increasing forward bias, the EL of the CT state becomes visible.

The data shown in Fig. S3 are normalized to the bulk-emission peak at 415 nm and recorded with a 400 nm long-pass filter (Thorlabs FELH0400) to exclude the excitation light. As apparent from Fig. S2, there is an increasing (though low) contribution of bulk EL with growing intrinsic layer thickness. This gives the impression that the CT-state PL decreases in Fig. S3D compared to S3C. Comparing the raw data, however, shows that the PL signal is on the same level.

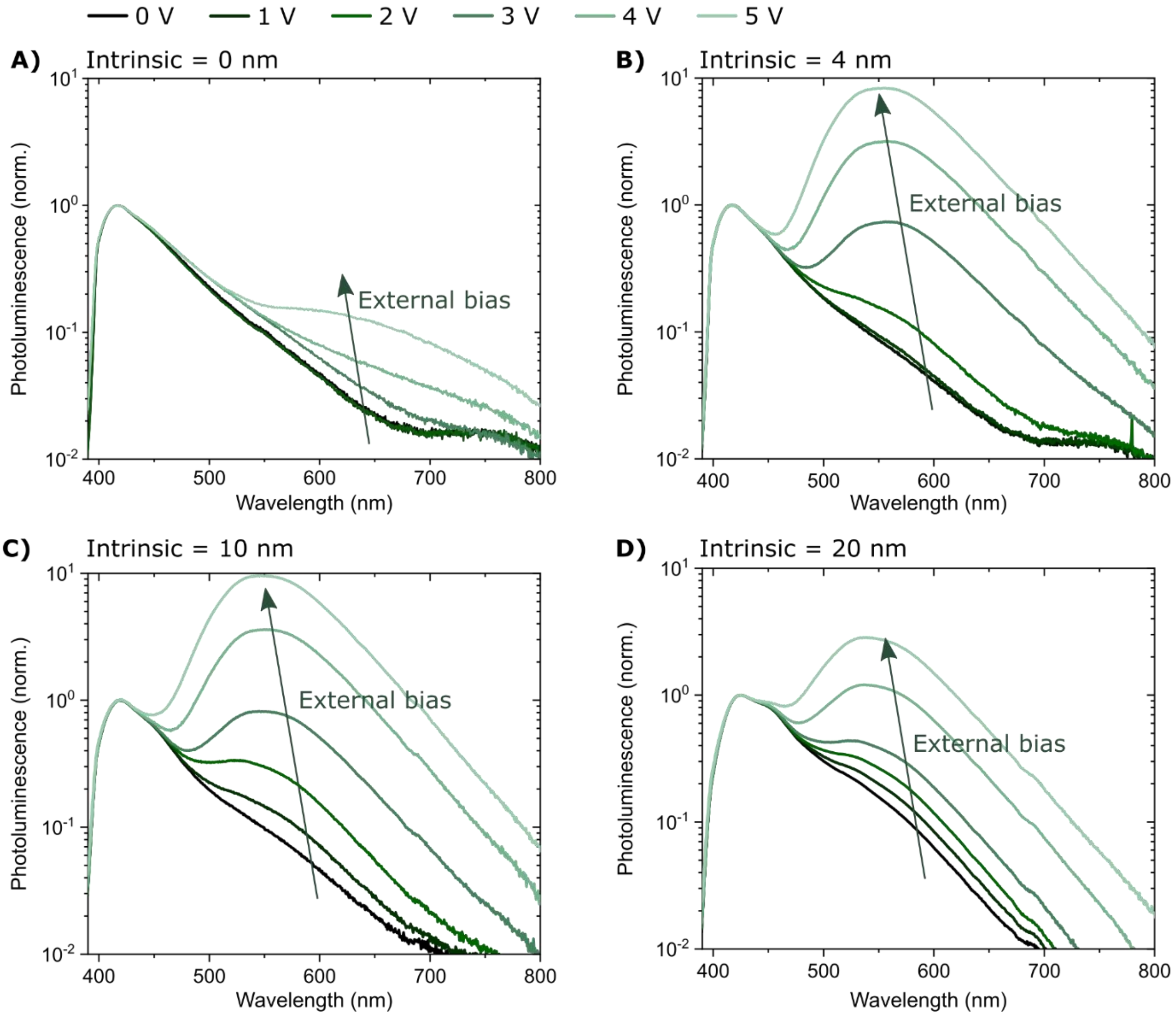

Figure S3. *Photoluminescence measurements of the investigated p-(i-)n samples at different external bias voltages using a mounted LED for 365 nm excitation and a 400 nm long-pass filter. The intrinsic layer thickness is A) 0 nm, B) 4 nm, C) 10 nm, and D) 20 nm. The data are normalized to the m-MTDATA emission at 415 nm.*

S4. Electroluminescence turn-on behavior

Using a photomultiplier tube (PMT, Hamamatsu C12918), the light-emission onset of the p-(i-)n samples studied in the main manuscript is investigated. The devices are driven by a Keithley 2400 SMU and the measurement is operated by the software SweepMe! (sweep-me.net). Compared to Fig. S1, where a calibrated silicon photodiode is used to track the light emission, the PMT provides a much higher sensitivity in the onset region. Figure S4 shows a shift to a higher onset voltage with increasing intrinsic layer thickness by approximately 100 mV. This could relate to an increasing CT-state energy (activation energy) but also to an increasing series resistance of the devices with increasing intrinsic layer thickness.

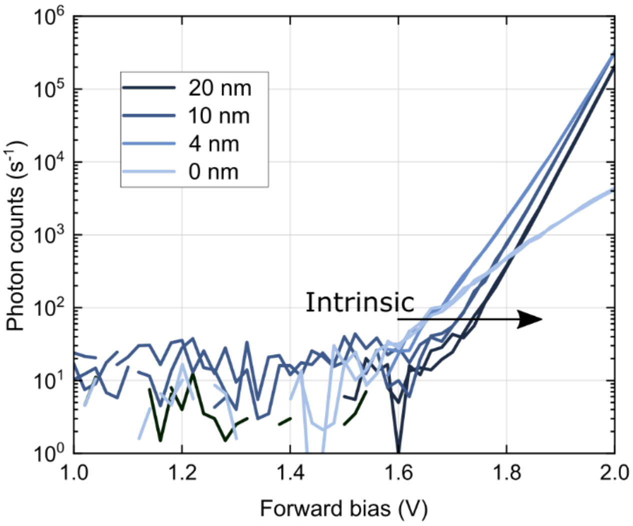

Figure S4. *Turn-on behavior of devices with increasing intrinsic layer thickness measured with a PMT in forward and backward direction.*

S5. Photovoltaic characterization

The photovoltaic characteristics of the p-(i-)n samples studied in the main manuscript are investigated. The open-circuit voltage V_{OC}, which marks a lower limit for E_{CT}, increases with intrinsic layer thickness. This may be related to an increasing E_{CT} and a decrease in non-radiative voltage losses, cf. Tab S1.

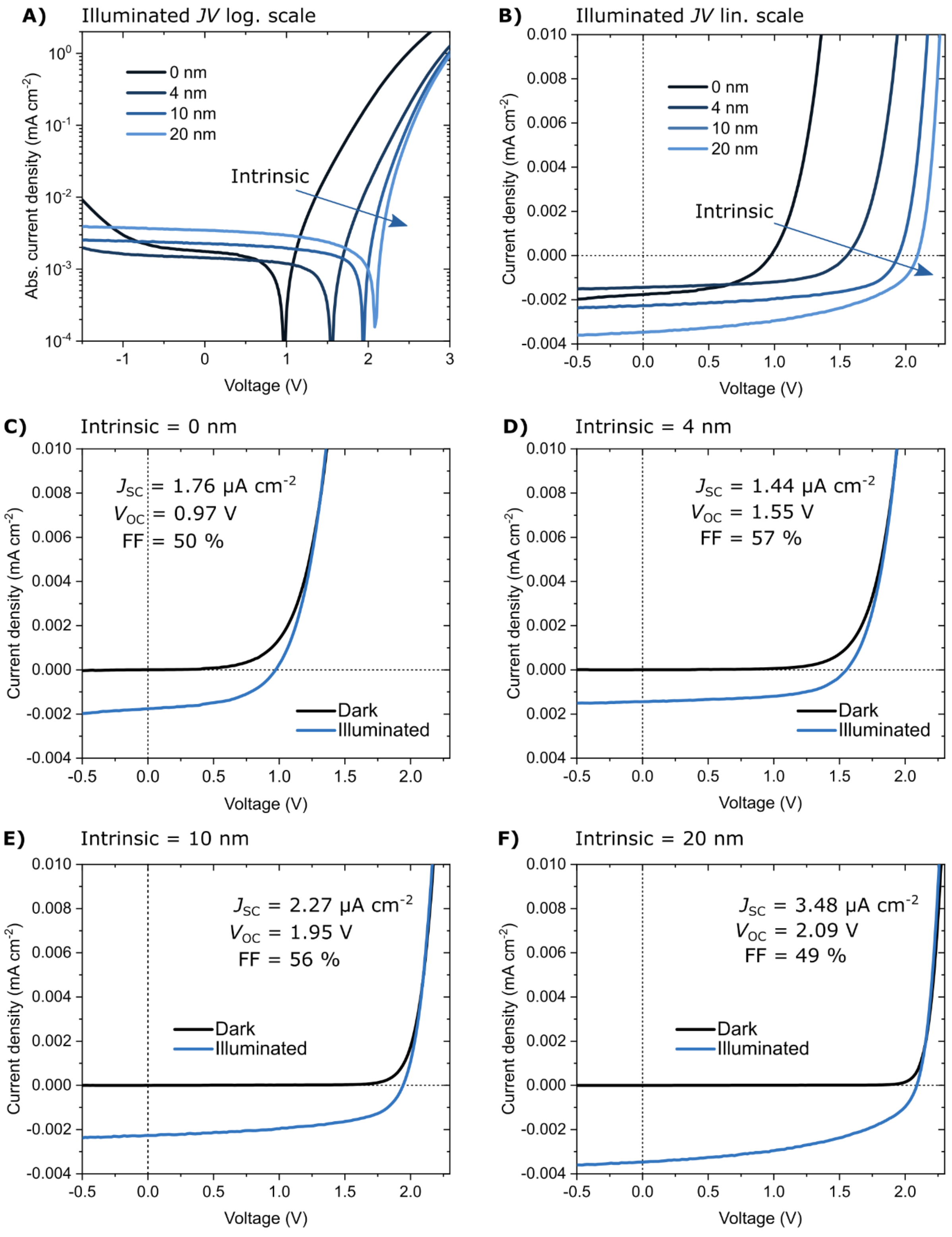

Figure S5. *Illuminated current density-voltage (J-V) characterization for all investigated samples in A) logarithmic and B) linear scale. J-V curves for C) 0 nm, D) 4 nm, E) 10 nm, and F) 20 nm of intrinsic layer thicknesses under illumination and in the dark. Illumination corresponds to 100 mW m*$^{-2}$ *without applying a background correction.*

Table S1. *Measured EQE_{EL} values at 1 mA and calculated non-radiative voltage losses (ΔV_{nr}).*

	0 nm	4 nm	10 nm	20 nm
EQE_{EL} [%]	$4 \cdot 10^{-4}$	0.6	1.3	1.5
ΔV_{nr} [V]	0.3	0.1	0.1	0.1

Table S1 presents an estimation of the non-radiative voltage losses in our devices. They are calculated by:

$$\Delta V_{\mathrm{nr}} = \frac{k_{\mathrm{B}}T}{e} \ln\left(\frac{1}{EQE_{\mathrm{EL}}}\right) \quad (1)\,,$$

with Boltzmann constant k_B, elementary charge e, and temperature T = 293 K (cf. Benduhn *et al.*, *Nat. Energy*, 2017, https://doi.org/10.1038/nenergy.2017.53).

Illuminated current–voltage characteristics are performed at an incident light intensity of 100 mW cm^{-2}. The light is generated by a sun simulator (Solar Light Co. Sunlight simulator 16S-003-300-AM1.5, USA) utilizing a 300 W xenon lamp (Ushio UXL-300D-0, Japan). The intensity is calibrated to a silicon photodiode (Hamamatsu Photonics S1337, Japan), but no mismatch correction was applied. The voltage-dependent current is measured by an SMU (Keithley Instruments SMU 2400, USA). The setup is controlled by the measurement software SweepMe! (sweep-me.net).

S6. UV-vis absorption spectroscopy

To further characterize the material system studied in the main manuscript, the absorptance of individual and blended films, evaporated onto glass substrates, is investigated using a Shimadzu SolidSpec-3700 UV-vis-NIR absorption spectrometer. The absorptance is $A = 1 - T - R$, with T being transmission and R reflection taken in an integrating sphere, and is corrected against a glass sample.

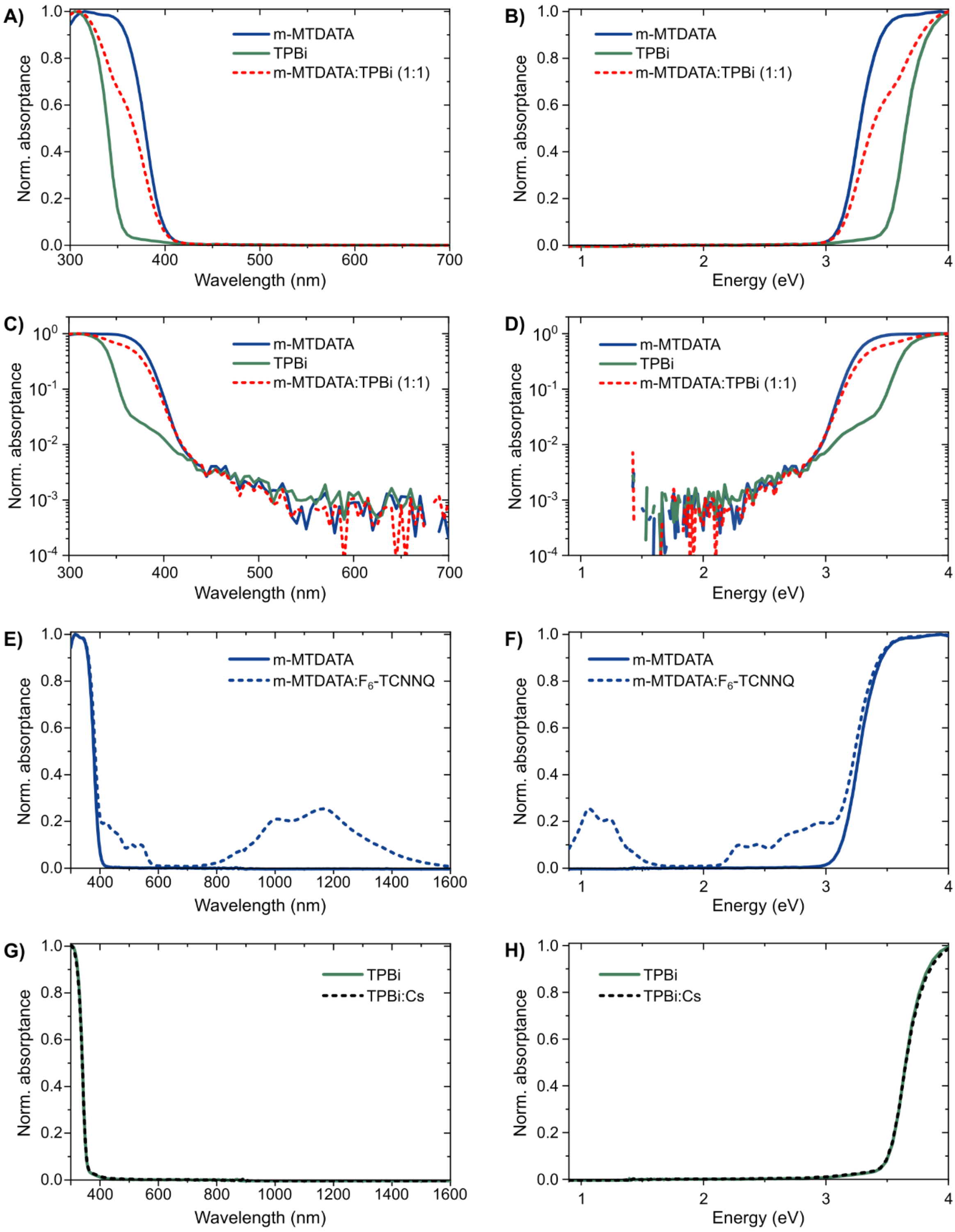

Figure S6. *UV-vis absorption spectroscopy taken with evaporated layers on glass. A) - D): m-MTDATA, TPBi, and m-MTDATA:TPBi (co-evaporated 1:1), all 70 nm film thickness; E) + F): m-MTDATA (70 nm) and m-MTDATA:F_6-TCNNQ (4 wt%, 96 nm film thickness); G) + H): TPBi and TPBi:Cs (1:1), 70 nm film thickness.*

Figures S6A-S6D show the experiments with the bulk materials m-MTDATA and TPBi and the co-evaporated, blended (1:1) film. The results resemble the characteristics measured by dos Santos, Monkman, *et al*. (https://doi.org/10.1021/acs.jpcc.6b05198). In contrast to the planar heterojunction, which we use in the main manuscript, the co-evaporated film represents a bulk heterojunction. Even for this blend, we cannot identify a CT-state absorption feature, while F_6-TCNNQ induces a strong absorption in this region. This may indicate that the $sEQE_{PV}$ feature of the planar m-MTDATA/TPBi CT state in the main manuscript is obscured by the dopant-induced absorption.

Comparing the doped and undoped hole-transport layer, Figs. S6E and S6F show clear traces of F_6-TCNNQ (and radical) absorption just above 1 eV and around 2.5 eV. These characteristics are further investigated in the subsequent section. Finally, by comparing the doped and undoped electron-transport layers (Figs. S6G and S6H), no visible impact of Cs can be measured.

All spectra are normalized to the maximum value.

S7. Further spectroscopy on F_6-TCNNQ

Since F_6-TCNNQ seems to play a decisive role in the interpretation of our $sEQE_{PV}$ data, cf. Fig. 4 and Fig. 5 in the main article, we run two more experiments to understand its behavior.

First, we introduce it into a different host (Spiro-TAD, 2,2',7,7'-tetrakis(diphenylamino)-9,9'-spirobifluorene) and measure the absorbance of both the neat film (30 nm, thermal evaporation on a quartz glass substrate) and the blended film (120 nm, thermal co-evaporation, 20 wt% doping ratio) using again the Shimadzu SolidSpec-3700 UV-vis-NIR spectrometer. The absorbance is calculated from the sample's transmittance T as $A = -\log_{10}(T)$. The neat F_6-TCNNQ film shows an absorbance feature around 2.5 eV and the neat Spiro-TAD film absorbance beyond 3 eV. The blended film contains both contributions and an additional hump around 1 eV. It resembles the feature measured in the blend of m-MTDATA:F_6-TCNNQ, as presented in Fig. S6. Its shape appears slightly altered, which probably stems from different polarization influences in different host materials. As this feature around 1 eV is independent of the host material, it indicates anion radical absorption of the dopant molecule. This is further examined in the following spectroelectrochemistry experiment.

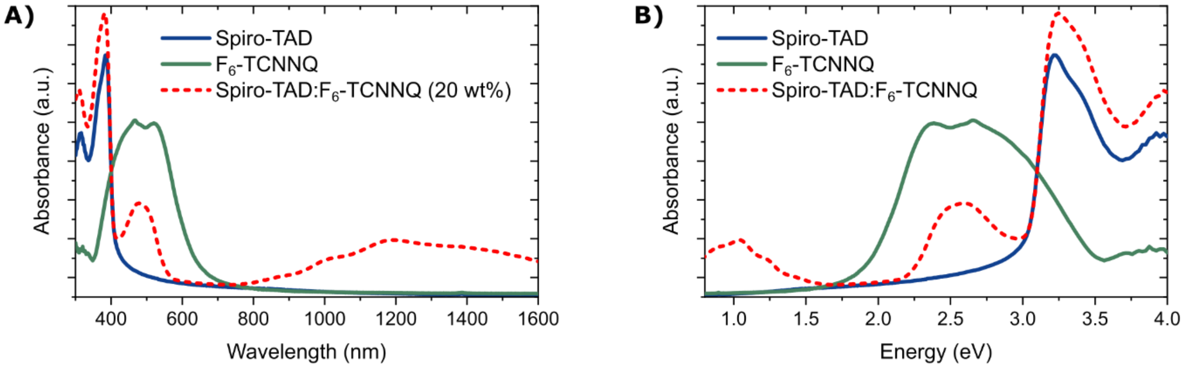

Figure S7. *Absorbance of Spiro-TAD (30 nm), F_6-TCNNQ (30 nm), and the blended film (120 nm, 20 wt%) over A) wavelength and B) energy. The absorbance characteristics are rescaled by an arbitrary factor in this figure to match the absorbance magnitude of the single-component films which have a smaller thickness.*

Second, to gather further evidence about the neutral and radical characteristics of F_6-TCNNQ, we run a cyclic voltammetry (CV) scan accompanied by transmission measurements using an Autolab Potentiostat Galvanostat (PGSTAT204), NOVA 2.1 as operating software, an AvaLight-DH-S-BAL (deuterium + halogene lamp combination) excitation source and AvaSpec-ULS204BCL-EVD (UV), AvaSpec-NIR (IR) as detection unit, operated by Avasoft 8.5.0.0. The electrodes are made of platinum (working electrode), graphite (counter electrode), and silver (reference electrode).

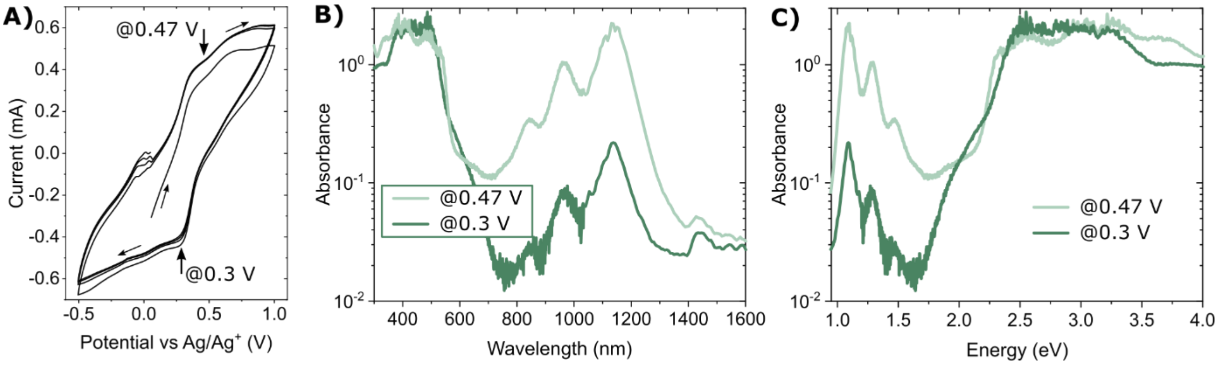

Figure S8. *A) Cyclic voltammetry measurement of F_6-TCNNQ using 0.1 mol/l nBu_4NPF_6 in acetonitrile (MeCN) as electrolyte. Absorbance spectra for the predominantly radical (@0.47 V) and predominantly neutral (@0.3 V) state over B) wavelength and C) energy.*

The CV scans shown in Fig. S8A are run at 25 mV/s, starting at 0.05 V, ramping the working-electrode bias up to 1.0 V, down to -0.5 V, up to 1.0 V again, completing 4 cycles, and finishing again at 0.05 V. At each voltage step, a transmission spectrum is taken which is automatically corrected by a previously acquired reference spectrum. The absorbance spectra in Fig. S8B and S8C present the same data over wavelength and energy. The spectrum taken at 0.30 V is acquired during the first downward scan, and the spectrum at 0.47 V at the third upward scan. The redox reaction of the system seems not entirely reversible but the magnitude of the double peak of interest at 1.1 eV and 1.3 eV alters significantly. At 0.47 V, we deduce from the CV scan, most F_6-TCNNQ molecules are reduced. Here, we can assume to predominantly measure the anion radical characteristics. They show a very prominent increase of the double (or even triple) absorbance feature above 1 eV. In the neutral state at 0.3 V, we presume that a minor fraction of molecules remains reduced, which gives rise to the still-existent but significantly lower (note the logarithmic y-axis) radical absorbance characteristics.

What mostly remains stable is the absorbance around 2.5 eV, which was also visible in Figs. S6 and S7 and most likely mainly forms the hump around 2.5 eV in the $sEQE_{PV}$ spectra presented in the main manuscript, cf. Fig. 4 and 5.

S8. *sEQE*$_{PV}$ measurements under illumination

Here, we want to investigate if excitonic state-filling due to bias illumination may cause a shift in the CT absorption. In this experiment, we run *sEQE* measurements with lock-in technique while illuminating the system continuously with the indicated spectra, using either a 340 nm (Thorlabs, M340L4) or white (Thorlabs, MWWHLP1) mounted LED at different operating currents. At 0.7 A, the LEDs produce an optical power density of roughly 6 suns (white) or 0.5 suns (340 nm).

A clear illumination-dependent shift cannot be observed from the original *sEQE* data, cf. Figs. S9A and S9C. From the relative changes shown in Figs. S9B and S9D, one can speculate to identify shifting features around 1.6 eV and 2.7 eV under increasing illumination. This could correspond to a state-filling effect. We are, however, not able to verify this, and the respective CT absorption feature suffers potentially again from a superposition with the F_6-TCNNQ feature.

Also, the shift appears at a very low photocurrent signal while the sample is exposed to intense background illumination (about 6 suns for white light at 0.7 A) causing a high offset photocurrent. We are careful not to overinterpret the data.

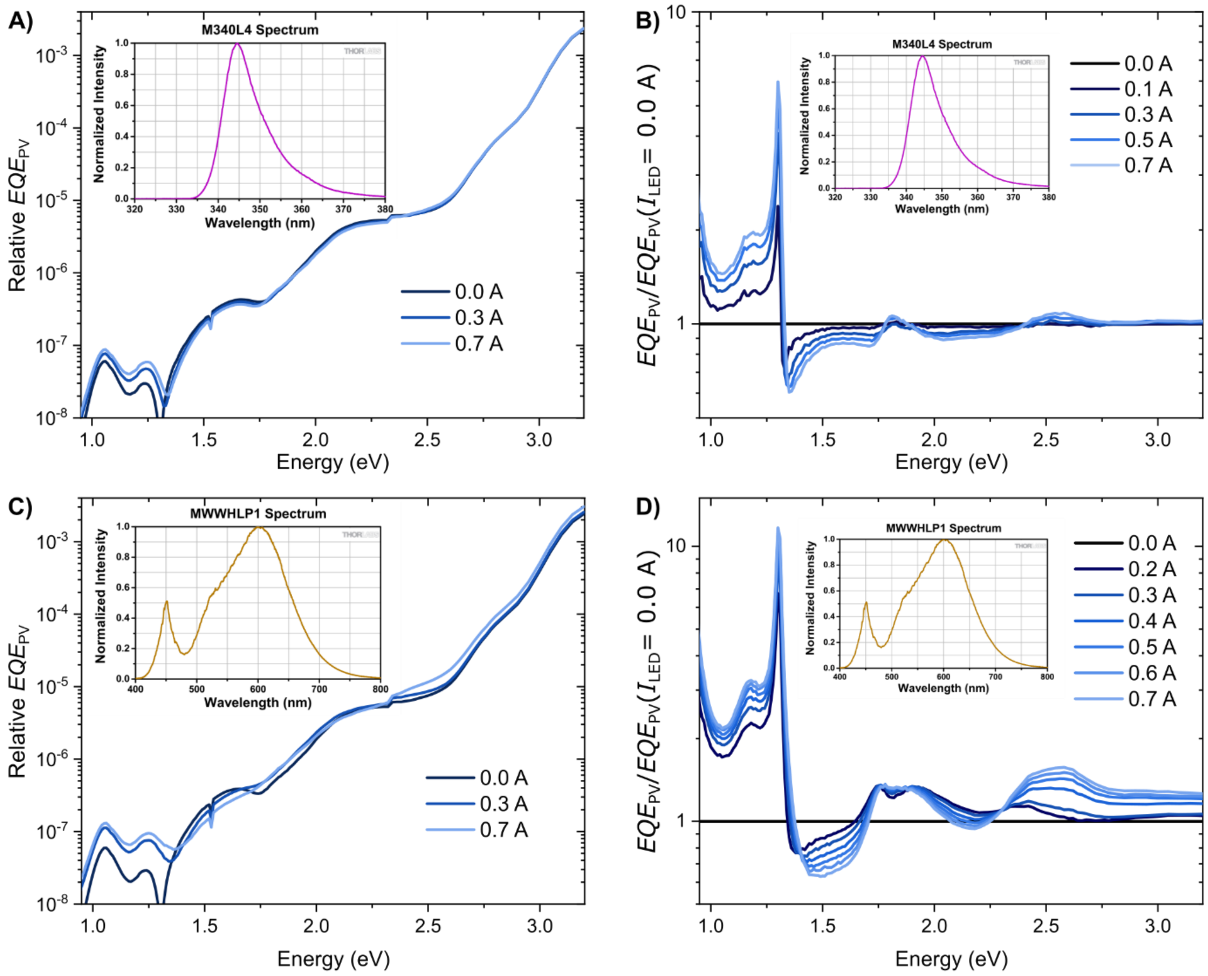

Figure S9. *sEQE measurements under increasing bias illumination with A) a 340 nm LED and C) a white LED using the p-n (m-MTDATA/TPBi) system presented in the main manuscript under short-circuit conditions. Panels B) and D) indicate the relative changes to the unilluminated case. The legends indicate the LED driving current.*

S9. Asymmetric intrinsic layers

To study the impact of the dopants on the observed sEQE spectra, we fabricate samples with intrinsic layers either on the electron- or hole-transporting side. The overall thickness of the devices and the thickness of both transport layers remain stable, cf. Fig. S10A and S10B. Two trends can be observed:

First, the double feature just above 1 eV remains clearly visible when removing the Cs from the interface (Fig. S10A) but vanishes when taking the F_6-TCNNQ away (Fig. S10B). This proves that this feature corresponds to the p-dopant, as investigated in Section S7.

Second, the shoulder at 2.8 eV decreases or blue-shifts rapidly in both cases, when removing Cs or F_6-TCNNQ from the interface. This could imply an impeded exciton splitting at the interface since the energy level bending is reduced. It could also mean that E_{CT} is shifted according to our hypothesis.

Interestingly, there is also a distinct difference in the bulk signal between both scenarios. When removing the p-dopant from the interface, cf. Fig. S10B, the m-MTDATA feature around 3.1 eV rises, potentially due to an increase in the share of neutral m-MTDATA molecules close to the interface, while the remainder of the spectrum decreases slightly. In the case of Fig. S10A, the whole spectrum decreases slightly with removing Cs from the interface, potentially due to a lower intrinsic electric field at the interface and hence reduced exciton-splitting efficiency.

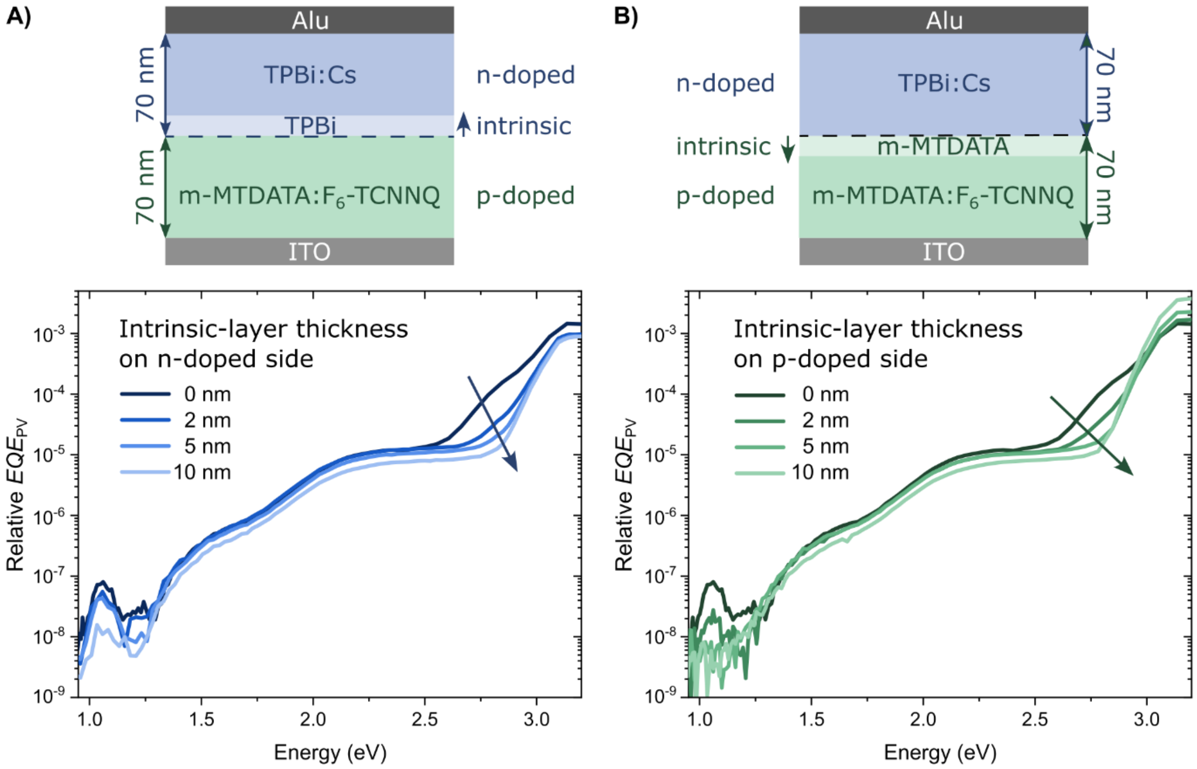

__Figure S10.__ Introducing intrinsic layers only at A) the n-doped or B) the p-doped side provides more information about the sEQE features around 1.1 eV and 2.7 eV.

S10. Further material systems

A) m-MTDATA:F_6-TCNNQ (10 wt%) / TPBi: $W_2(hpp)_4$ (10 wt%)

Here, we vary the original material combination by changing the n-dopant to a molecular species and by increasing the doping concentration to further reduce the series resistances in the device. Unfortunately, this yields extremely high leakage currents, and the devices emit no detectable light.

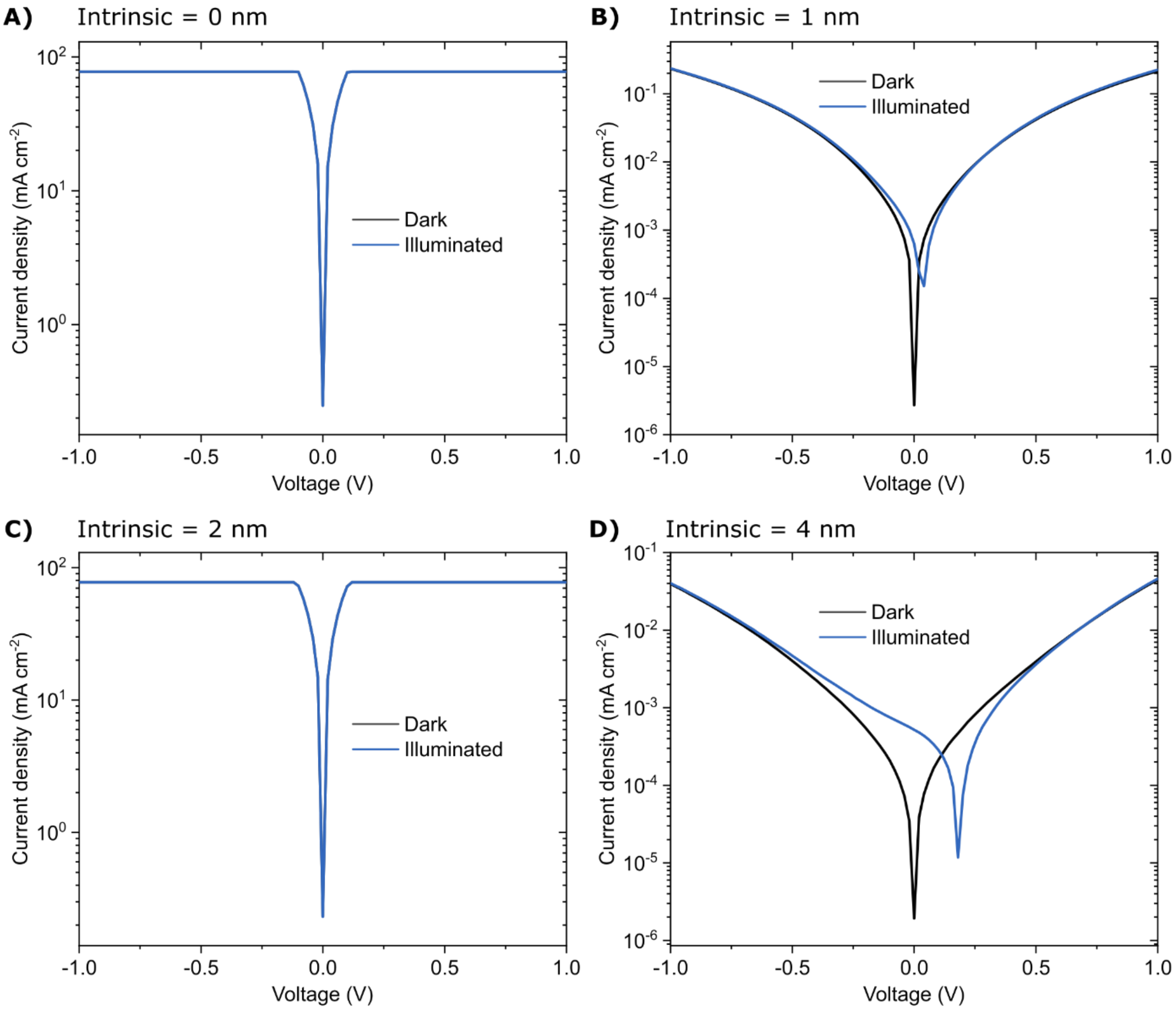

Figure S11. *JV curves of the introduced stack with intrinsic layer thicknesses of A) 0 nm, B) 1 nm, C) 2 nm, and D) 4 nm under illumination (100 mW m^{-2}) and in the dark.*

B) BF-DBP:F_6-TCNNQ (2 wt%) / B4PYMPM:$W_2(hpp)_4$ (2 wt%)

For the sake of generality, we test a second material system, which is more relevant for photovoltaic applications. We have previously reported that it can be employed for both charge-generating and light-emitting applications (Ullbrich *et al*, Nat. Materials, 2019, https://doi.org/10.1038/s41563-019-0324-5). The materials employed are N,N'-[(Diphenyl-N,N'-bis)9,9,-dimethyl-fluoren-2-yl]-benzidine (BF-DBP), Bis-4,6-(3,5-di-4-pyridylphenyl)-2-methylpyrimidine (B4PYMPM), and ditungsten tetra(hpp) ($W_2(hpp)_4$). The stack is structured as follows:

Ag (100 nm) / BF-DBP:F_6-TCNNQ (2 wt%, 70 nm) / B4PYMPM:$W_2(hpp)_4$ (2 wt%, 70 nm) / ITO

And again, we introduced a varying intrinsic layer thickness (here, from 0 to 4 nm) at the interface.

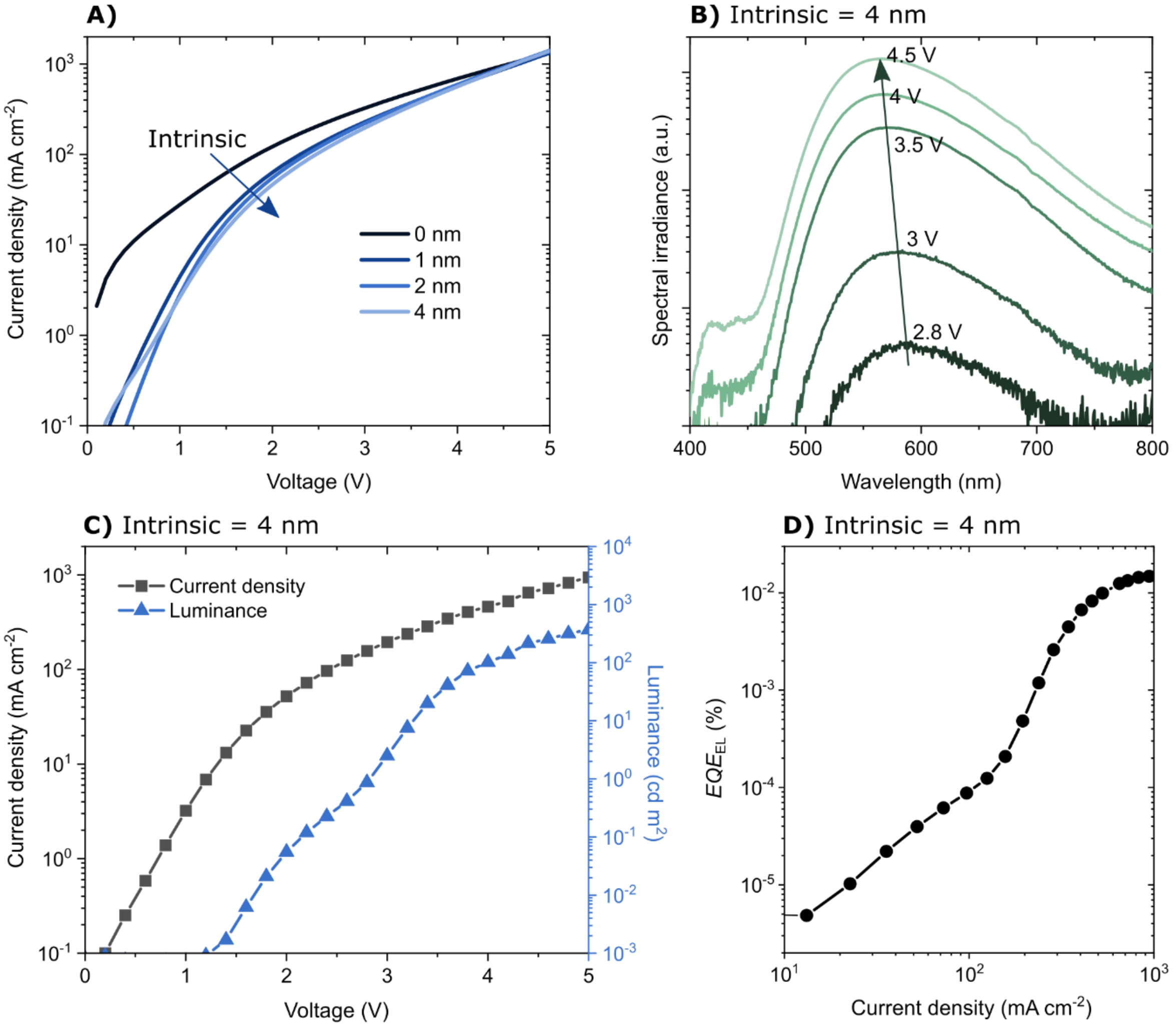

***Figure S12.** Characteristics for another material system with a BF-DBP/B4PYMPM (2wt % doping) interface CT state. A) The leakage currents are much more pronounced than in the m-MTDATA/TPBi system, even for increasing intrinsic layers. B) The shift in EL is again visible, C) the luminance is much weaker D) and hence the EQE_{EL} significantly lower.*

Figure S12 shows some main characteristics of the devices. Even with 4 nm of intrinsic layer at the interface (2 nm on each side), the system yields very high leakage currents, cf. Fig. S12A. The samples with an even thinner intrinsic layer do not show any detectable photon emission at all. So, the intrinsic-layer dependent EL shift cannot be investigated. The EL shift with voltage can be detected for the $i = 4$ nm sample, cf. Fig. S12B, but is less pronounced than in the material system presented in the main manuscript.

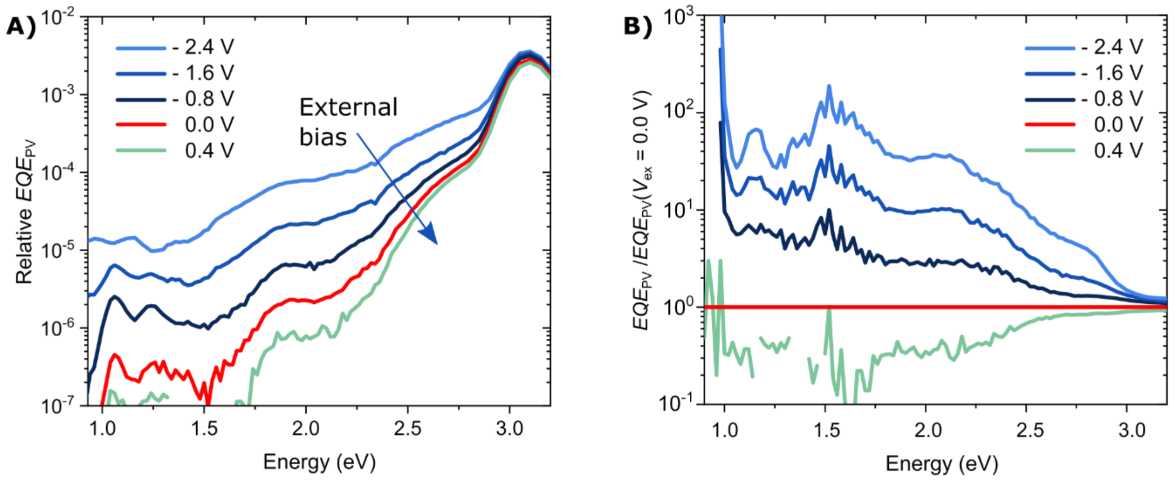

Figure S13. *A) sEQE characteristics and B) relative change to short-circuit conditions for the BF-DBP/B4PYMPM (2 nm intrinsic layer thickness) system.*

The *sEQE* measurements, cf. Fig. S13, do not show a clear voltage-dependent lateral shift around 2.7 eV. The below-bulk *sEQE* signal increases with negative bias, the bulk absorption is clearly visible, and the F_6-TCNNQ radical feature is once more apparent. The feature around 2 eV may again be caused by the dopant.

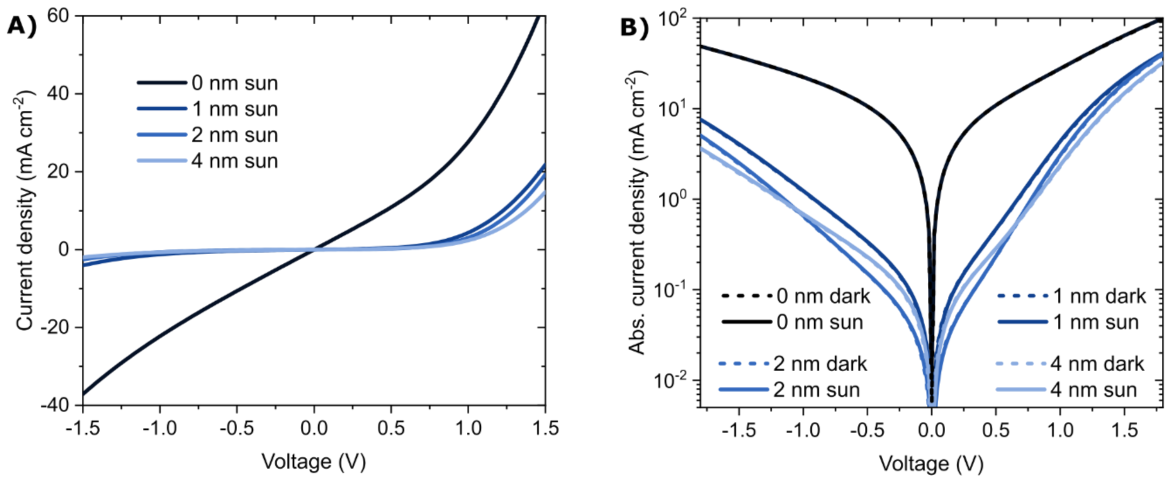

Figure S14. *Current density-voltage curves of the BF-DBP/B4PYMPM system for different intrinsic layer thicknesses in A) linear and B) logarithmic scale under illumination (100mW cm^{-2}) and in darkness. The leakage currents are too high to measure open-circuit voltages.*

When characterizing the system in terms of photovoltaic parameters, the leakage currents are too high to yield any reasonable result. We can conclude that the system is not suitable for building organic p-n junctions and suffers from too high leakage currents to provide conclusive results.

S11. Calculation of depletion width, doping concentration, and intrinsic electric field

This assessment follows the understanding of classical semiconductor p-n junctions including the assumptions of the Shockley equation, i.e. no charge carriers in the depletion layer and no electron-electron interaction. It is therefore expected to fail at elevated forward bias and is only meant to roughly estimate the electric fields at the p-n interface.

The estimation of the ionized acceptor concentration in m-MTDATA:F_6-TCNNQ (4 wt%) follows the assessment in Kirch *et al.*, Phys. Rev. Appl., 2022 (https://doi.org/10.1103/PhysRevApplied.18.034017) and yields $N_A \approx 8 \cdot 10^{18}$ cm^{-3}.

On the n-doped side, the ionized donor concentration N_D is not as simple to determine, as we cannot monitor the Cs evaporation rate using the quartz crystal microbalance incorporated in the evaporation chamber. Instead, the evaporation rates are determined by an ETL conductivity test and may yield varying molar ratios between batches. From experience, a molar ratio TPBi:Cs of roughly 1:1 can be assumed. The doping efficiency and oxidation of cesium atoms reduce the final yield of ionized atoms, i.e. polarons, in a very uncertain way. Thus, we need a different estimation basis.

In previous studies from our lab, the depletion layer W_n of related material systems was estimated to range below 3 nm (Kleemann *et al.* Nano Lett. 2010, https://doi.org/10.1021/nl102916n). If we take this value as a benchmark (it may be smaller corresponding to even higher fields), this corresponds to an ionized donor concentration of about $N_D \approx 2 \cdot 10^{19}$ cm^{-3}. At zero bias and no intrinsic layer, the depletion regions *W* can thus be calculated as follows:

$$W_p = \sqrt{\frac{2\epsilon_0\epsilon_r\Psi_{BI}\,N_D}{e\,N_A(N_A+N_D)}} \approx 9\text{ nm} \quad (2)$$

$$W_n = \sqrt{\frac{2\epsilon_0\epsilon_r\Psi_{BI}\,N_A}{e\,N_D(N_A+N_D)}} \approx 3\text{ nm} \quad (3).$$

The built-in potential Ψ_{BI} for doped layers is slightly lower than the difference IE_D - EA_A = 2.4 eV, as the Fermi levels approach the respective HOMO and LUMO levels, and is assumed to be roughly 2 eV. The relative permittivity is assumed as $\varepsilon_r \approx 3.5$, ε_0 is the dielectric constant, and *e* is the elementary charge.

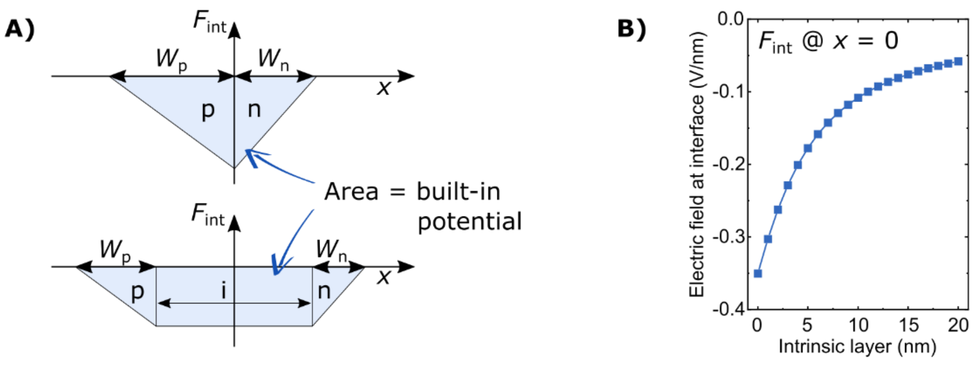

Figure S15. *Illustration of the assessment for intrinsic electric field magnitudes: A) Intrinsic electric field in a p-(i-)n junction and B) its magnitude at the interface x = 0.*

Now, we insert an intrinsic layer between the p-doped and n-doped regions. To evaluate the electric field at the interface (x = 0), we must find the respective depletion regions. The integral of the electric field must always yield Ψ_{BI}, no matter what intrinsic layer thickness i is inserted:

$$\Psi_{\mathrm{BI}} = \frac{e\, N_{\mathrm{A}}\left(W_{\mathrm{p}} - i\right)}{\epsilon_0 \epsilon_{\mathrm{r}}}\left(2i - \frac{W_{\mathrm{p}} - i}{2} + \frac{W_n - i}{2}\right) \quad (4).$$

With $W_n = \frac{N_{\mathrm{A}}}{N_{\mathrm{D}}} W_{\mathrm{p}}$, one can solve this equation for $W_{\mathrm{p}} = W_{\mathrm{p}}(i)$. The solution can be obtained analytically, here we used the Python solver *sympy* to calculate the resulting intrinsic electric field at the interface:

$$F_{\mathrm{int}}(x = 0) = \frac{-e\, N_{\mathrm{A}} W_{\mathrm{p}}}{\epsilon_0 \epsilon_{\mathrm{r}}} = F_{\mathrm{max}}(i) \quad (5).$$

The graphical solution to this equation is plotted in the main manuscript, Fig. 3B, and here Fig. S15.

S12. Modeling of the p-n junction with Setfos

The Setfos drift-diffusion model uses the same parameters as estimated in the previous section, again listed in Table S2. The Setfos parameter file ("p-i-n junction.parx") is available as a supplementary file. Further material parameters like electron and hole mobility, and relative permittivity are taken from the Setfos material database. The temperature is set to 300 K. The layer thicknesses are set according to the experiments.

Table S2. *List of parameters used for drift-diffusion modeling.*

**Parameter**	**Value**
m-MTDATA HOMO level	5.1 eV
m-MTDATA LUMO level	1.9 eV
m-MTDATA DOS HOMO and LUMO	10^{21} cm^{-3}
Acceptor doping concentration	$8{\cdot}10^{18}$ cm^{-3}
TPBi HOMO level	6.2 eV
TPBi LUMO level	2.7 eV
TPBi DOS HOMO and LUMO	10^{21} cm^{-3}
Donor doping concentration	$2{\cdot}10^{19}$ cm^{-3}
Bulk recombination	Langevin
Interface recombination	Shockley-Read-Hall
m-MTDATA electron zero-field mobility	$4.79{\cdot}10^{-8}$ cm^2/V/s
m-MTDATA hole zero-field mobility	$4.79{\cdot}10^{-6}$ cm^2/V/s
m-MTDATA gamma mobility	0.0034 (cm/V)$^{0.5}$
TPBi electron zero-field mobility	$8.53{\cdot}10^{-7}$ cm^2/V/s
TPBi hole zero-field mobility	$8.53{\cdot}10^{-6}$ cm^2/V/s
TPBi gamma mobility	0.00085 (cm/V)$^{0.5}$

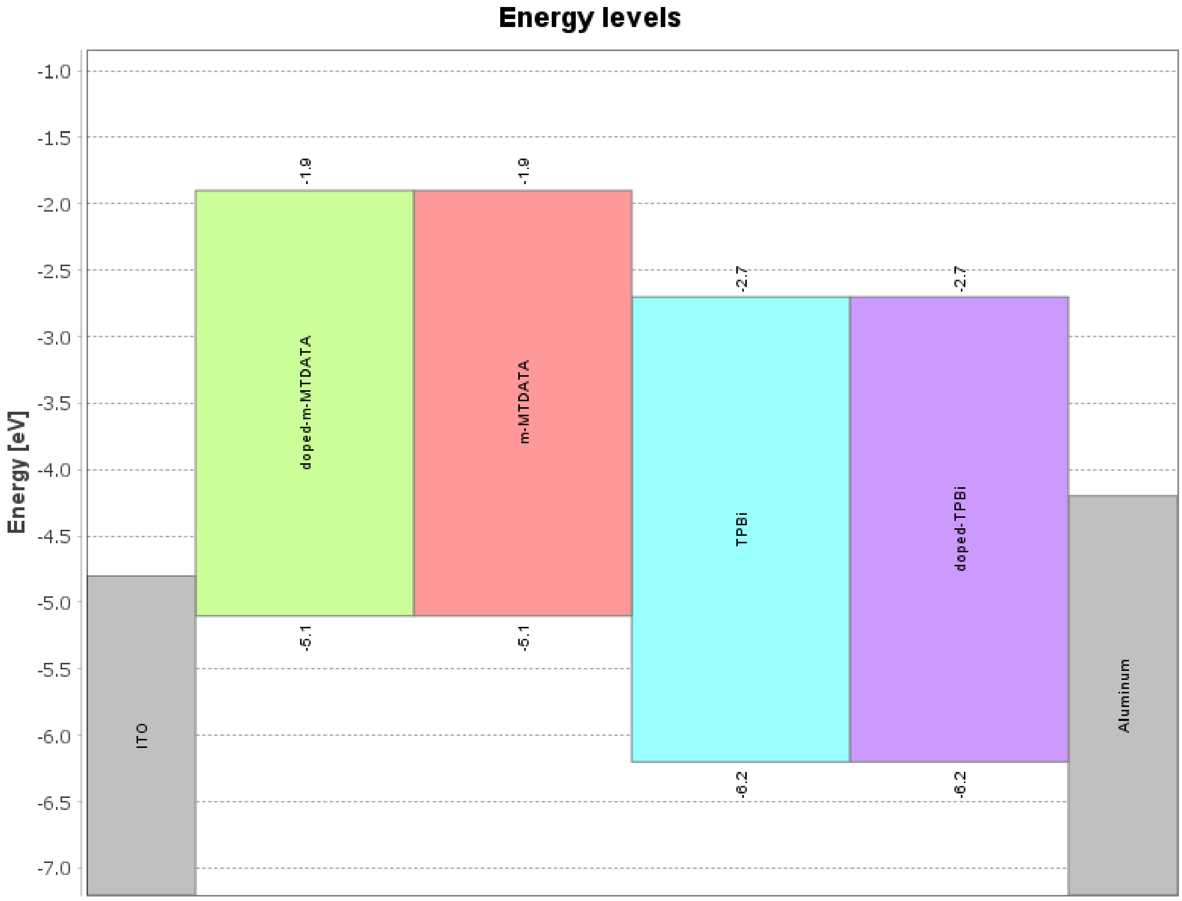

Figure S16. *Stack sketch and energy level diagram as used in the Setfos model. Layer thicknesses are not drawn to scale.*

S13. Comparison to randomly oriented dipoles in a bulk heterojunction

Here, we compare the voltage-dependent emission and absorption of the planar heterojunction (PHJ) measured in the main manuscript with a device featuring a bulk heterojunction (BHJ).

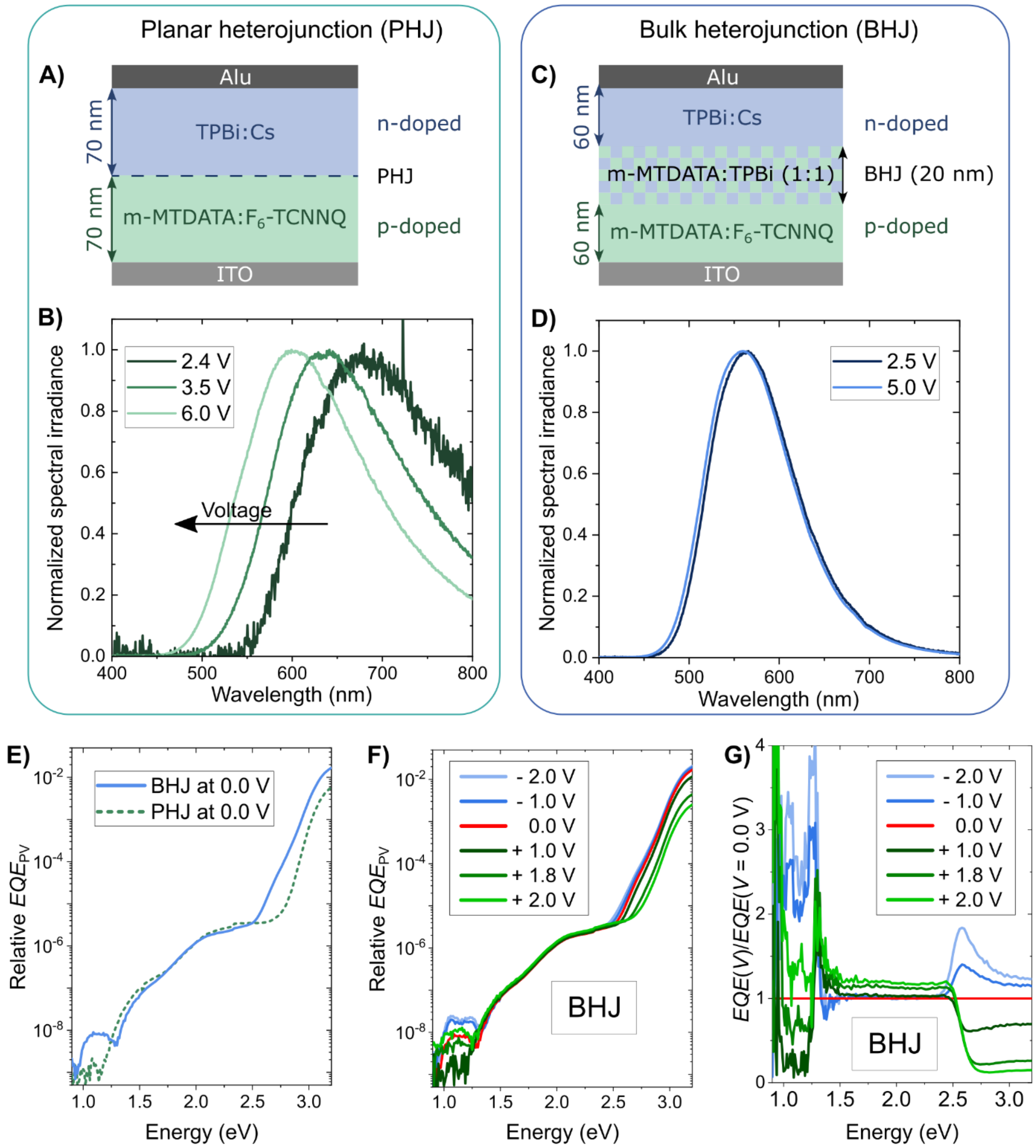

__Figure S17.__ Comparison between the voltage-dependent EL of a planar heterojunction (PHJ) in A) and B) and a bulk heterojunction (BHJ) in C) and D). Panel E) compares the sEQE$_{PV}$ characteristics of the BHJ and a PHJ with 20 nm of intrinsic layers in the middle, F) the voltage-dependent sEQE$_{PV}$ of the BHJ, and G) the voltage-dependent sEQE$_{PV}$ change in F) relative to the short-circuit condition.

As shown in Fig. S17C, the BHJ device has two doped transport layers to ensure efficient charge-carrier injection and transport. In the middle, we introduce 20 nm of co-evaporated m-MTDATA:TPBi (1:1) instead of the PHJ presented in the main manuscript, cf. Fig. S17A.

While, according to the reasoning given in the main manuscript, the PHJ exhibits directed CT dipoles at the interface which are prone to be tuned by interface electric fields, the dipoles in the BHJ are randomly oriented. Under increasing forward bias, as shown in Fig. S17B and D, the BHJ device does not show a significant shift of the emission wavelength.

In Fig. S17E, a PHJ device featuring 20 nm of intrinsic D-A layers in the middle (just as the i = 20 nm device in the main text) is compared with a device sporting 20 nm BHJ in the middle, cf. Fig. S17C. Due to the increased interface of intrinsic m-MTDATA and TPBi, we observe a higher bulk feature around 3.2eV (region (I), see main text for the definition of regions I to V). Additionally, the feature in region (II) is more pronounced which we attribute to the increased cross-section of CT-states between m-MTDATA and TPBi. Region (III) and (IV) are similar for both device architectures. We take this as an indication that these subgap features are governed by the transport layers and their interfaces to the intrinsic layers. The anionic F_6-TCNNQ feature is observable for the BHJ due to the interface of F_6-TCNNQ (in the HTL) and TPBi in the BHJ.

For the bias-dependent *sEQE* measurement of the BHJ device in Fig. S17F, regions (I) and (II) generate higher signal for reverse bias. This can be attributed to a higher charge-carrier extraction yield. Comparing these characteristics to the short-circuit condition in Fig. S17G, regions (III) and (IV) show an opposite trend compared to regions (I) and (II). We take this as a further indication that the nature of the absorption pathways in regions (III) and (IV) is fundamentally different and that the CT feature occurs in region (II).
